# Targeting Members of the Chemokine Family as a Novel Approach to Treating Neuropathic Pain

**DOI:** 10.3390/molecules28155766

**Published:** 2023-07-30

**Authors:** Katarzyna Pawlik, Joanna Mika

**Affiliations:** Department of Pain Pharmacology, Maj Institute of Pharmacology Polish Academy of Sciences, 12 Smetna Str., 31-343 Cracow, Poland; pawlik@if-pan.krakow.pl

**Keywords:** chemokines, chemokine receptors, neuropathic pain, bindarit, antagonist, agonist

## Abstract

Neuropathic pain is a debilitating condition that affects millions of people worldwide. Numerous studies indicate that this type of pain is a chronic condition with a complex mechanism that tends to worsen over time, leading to a significant deterioration in patients’ quality of life and issues like depression, disability, and disturbed sleep. Presently used analgesics are not effective enough in neuropathy treatment and may cause many side effects due to the high doses needed. In recent years, many researchers have pointed to the important role of chemokines not only in the development and maintenance of neuropathy but also in the effectiveness of analgesic drugs. Currently, approximately 50 chemokines are known to act through 20 different seven-transmembrane G-protein-coupled receptors located on the surface of neuronal, glial, and immune cells. Data from recent years clearly indicate that more chemokines than initially thought (CCL1/2/3/5/7/8/9/11, CXCL3/9/10/12/13/14/17; XCL1, CX3CL1) have pronociceptive properties; therefore, blocking their action by using neutralizing antibodies, inhibiting their synthesis, or blocking their receptors brings neuropathic pain relief. Several of them (CCL1/2/3/7/9/XCL1) have been shown to be able to reduce opioid drug effectiveness in neuropathy, and neutralizing antibodies against them can restore morphine and/or buprenorphine analgesia. The latest research provides irrefutable evidence that chemokine receptors are promising targets for pharmacotherapy; chemokine receptor antagonists can relieve pain of different etiologies, and most of them are able to enhance opioid analgesia, for example, the blockade of CCR1 (J113863), CCR2 (RS504393), CCR3 (SB328437), CCR4 (C021), CCR5 (maraviroc/AZD5672/TAK-220), CXCR2 (NVPCXCR220/SB225002), CXCR3 (NBI-74330/AMG487), CXCR4 (AMD3100/AMD3465), and XCR1 (vMIP-II). Recent research has shown that multitarget antagonists of chemokine receptors, such as CCR2/5 (cenicriviroc), CXCR1/2 (reparixin), and CCR2/CCR5/CCR8 (RAP-103), are also very effective painkillers. A multidirectional strategy based on the modulation of neuronal–glial–immune interactions by changing the activity of the chemokine family can significantly improve the quality of life of patients suffering from neuropathic pain. However, members of the chemokine family are still underestimated pharmacological targets for pain treatment. In this article, we review the literature and provide new insights into the role of chemokines and their receptors in neuropathic pain.

## 1. Chemokines and Neuropathic Pain

Neuropathic pain is usually chronic, with an incidence ranging from 6.9% up to 10% of the general population, and is still an important clinical problem [1,2]. Diseases and injuries that involve the somatosensory nervous system may not only lead to a loss of its function but also increase hypersensitivity and evoke spontaneous pain [2]. Pain can result from etiologically diverse disorders affecting both peripheral and central nervous systems. The cause can be a metabolic, neurodegenerative, vascular, or autoimmune disease; a tumor; trauma; infection; or exposure to toxins. Neuropathic pain evokes severe suffering, disability, depression, and sleep disturbances [3,4,5], and its therapeutic management is challenging [6]. Recommended analgesics provide poor or unsatisfactory relief to patients [7]. The mechanism of neuropathic pain is complex, and its relationship with the pathological disease process often remains unclear [1,2]. The mechanism of neuropathic pain of various etiologies remains to be elucidated. Recently, an increasing number of studies have suggested the significant role of the development and maintenance of cytokines, especially chemokines. The knowledge of the function of individual chemokines will provide a better insight into the mechanisms of neuropathic pain and enable better therapy [8]. Today, approximately 50 chemokines are known (Table 1), which exert their effects via 20 different seven-transmembrane G-protein-coupled receptors localized on the surfaces of various immune and nervous cells [9,10]. Unfortunately, the chemokine family terminology is complicated since a double nomenclature is used, as a molecule’s name may refer to its biological function (e.g., Macrophage Inflammatory Protein-1 α (MIP-1α)) or to the setting of cysteine residues in the molecule plus a number (e.g., C-C motif chemokine ligand 2 (CCL2)) [11,12].

In 2000, Zlotnik and Yoshie introduced terminology that divides the chemokine family into receptors (R) and ligands (L) [47]. Currently, chemokines are named with the prefix CCL-, CXCL-, XCL-, or CX3CL-, and their receptors are analogously divided into four subfamilies, CCR-, CXCR-, XCR-, and CX3CR-. Notably, many chemokine receptors can be activated by several different ligands, e.g., CCL2/3/4/5/6/7/8/9-CCR1, CXCL1/2/3/5/6/7/8-CXCR2, but some of them are activated by only one specific chemokine, e.g., CX3CL1-CX3CR1 or CXCL16-CXCR6—more details in Table 2 Importantly, chemokines are produced not only by immune cells but also by neurons and glial cells and are responsible for the chemotaxis of many immune cells, including granulocytes, lymphocytes, and monocytes [48]. Chemokine production is increased in many neurodegenerative and neuroimmunological diseases, often accompanied by neuropathic pain [48,49]. The majority of chemokines detected in the central nervous system (CNS) are only expressed under pathological conditions, in contrast to their receptors, which are often constitutively expressed, such as CCR1, CCR2, CCR3, CCR5, CXCR2, CXCR3, CXCR4, and CX3CR1.

### 1.1. CC Chemokines in Neuropathic Pain

Numerous studies have indicated that there is a strong correlation between behavioral responses in various neuropathic pain models and changes in many chemokine receptors and their endogenous ligands. The largest group of chemokines is the CC subfamily, which consists of 28 members. These chemokines are produced by and attract many cells, including monocytes, neutrophils, eosinophils, basophils, T lymphocytes, natural killer cells, and dendritic cells [8,78]. In neuropathic pain caused by damage to the peripheral nervous system, especially by injury to the sciatic nerve, time-dependent changes in the levels of many endogenous chemokine receptor ligands have been shown in various anatomical structures in animals, including CCR1-CCL2, CCL3, CCL4, CCL5, CCL6, CCL7, CCL8, and CCL9 [16,53]; CCR2-CCL2 and CCL7 [15,52]; CCR3-CCL5, CCL7, CCL8, CCL11, CCL24, and CCL26 [16,23]; CCR4-CCL17 and CCL22 [27]; and CCR5-CCL3, CCL4, CCL5, CCL7, and CCL11 [56]. Some data unequivocally indicate that, in diabetic neuropathy, after nerve injury, time-dependent changes occur in some CC ligands: CCL2, CCL3, CCL4, and CCL9 [19,79]. Importantly, 11 chemokines from the CC group showed very strong pronociceptive properties after their intrathecal injection: CCL1 [13]; CCL2 [15,16]; CCL3 [16,19]; CCL4 [19]; CCL5 [16]; CCL7 [15,16]; CCL8 [16], CCL9 [16,19]; CCL17 [80]; CCL21 [28]; and CCL22 [80].

CCR1 seems to be one of the most important receptors involved in neuropathy because it is a target of several chemokines that have been revealed to possess strong pronociceptive properties in studies performed on rodents, such as CCL2, CCL3, CCL4, CCL5, CCL7, CCL8, and CCL9 [15,16,17,19,20], and CCL6 is still poorly studied for its role in nociceptive transmission. Importantly, CCR1 is also the receptor for CCL13, CCL14, CCL15, CCL16, and CCL23, but these chemokines are not present in mice and rats.

Several studies have shown that **CCL2** is one of the key factors upregulated during neuropathic pain development after nerve injury [16,51,53,81,82]. CCL2 acts as a ligand for CCR1, CCR2, and CCR4. Pharmacological studies have provided evidence that the intrathecal administration of CCL2 induces long-lasting pain-related behaviors in naive mice in a dose-dependent manner [15,17] and leads to microglial activation [51,83]. First, it was hypothesized that CCL2 is released by neurons and induces spinal microglia activation [51] and the phosphorylation of p38 MAPK [84], which leads to the production of numerous pronociceptive cytokines [85]. Later, it was discovered that activated astroglial cells are also a source of CCL2, which, after release, may activate CCR2 on spinal neurons and evoke sensitization by NMDA receptors through ERK pathway activation [15,17]. Currently, it is known that CCL2 can also be secreted by cells involved in neuropathy development, such as microglia, macrophages, and neutrophils [86,87,88]. Furthermore, the intrathecal injection of CCL2-neutralizing antibodies effectively reverses neuropathic pain-related behavior after nerve injury [15] and prevents microglial activation [51]. Moreover, CCL2 knockout by using siRNA may reduce pain-like behavior and macrophage density in DRGs after nerve crush [89]. Additionally, the inhibition of CCL2 in the spinal cord reduced mechanical hypersensitivity in a post-stroke animal model of neuropathy [90]. It is worth mentioning that the upregulation of this chemokine was also observed in neuropathy evoked by diabetes and chemotherapy [18,79]. These results highlight the pivotal role of CCL2 in nociception and suggest that it is an attractive target for the novel pharmacotherapy of neuropathic pain.

**CCL3** also appears to be an important factor in neuropathic pain development, as its injection induces hypersensitivity [16,20], and its pronociceptive properties are diminished by a CCR1 antagonist (BX513) [20]; however, this chemokine also acts as a ligand of CCR5. It was shown that after nerve injury at the spinal cord level, CCL3 is enhanced in parallel with microglial/macrophage cell activation [55]. CCL3 is secreted by numerous cell types, such as microglia, neurons, neutrophils, and T lymphocytes [91,92,93,94]. Importantly, although after nerve damage caused by CCI, the spinal mRNA level of *CCL3* is highly upregulated from the 2nd until the 28th day, its protein level is only changed between the 2nd and 7th days, suggesting the importance of CCL3 in the initial and middle phases of neuropathy [53,55]. Higher levels of this cytokine are also observed in diabetic neuropathy [19], chemotherapy-induced neuropathic pain [22,95], and a model of partial sciatic nerve ligation [21]. Notably, the neutralization of CCL3 reduces pain-like behavior development in STZ- [19], paclitaxel- [22], CCI- [57], and PSNL-induced [21] neuropathy in mice, which may indicate its significant role in this pathology. Importantly, it is postulated that autoantibodies against CCL3 are biomarkers of type 1 diabetes development [96]; therefore, research on the impact of the modulation of this chemokine on the development of neuropathy should undoubtedly be continued due to its potential therapeutic benefits.

**CCL4** participation in the development of neuropathic pain does not seem to be crucial, although it acts as a ligand of three chemokine receptors—CCR1, CCR5, and CCR8. Recently, in vitro studies have suggested that microglia and astroglia might be CCL4-producing cells, suggesting that this chemokine may be involved in nociception [56]. To date, only the mRNA level of *CCL4* has been shown to be increased after peripheral nerve injury in the spinal cord [16,19,53], while a protein increase has not been observed [16,57]. Similarly, studies conducted in streptozotocin- and chemotherapy-induced neuropathy models have suggested little, if any, participation of CCL4 in pathological nociception [19,97]. These data suggest that CCL4 may not be an appropriate target for future therapies for neuropathic pain.

**CCL5** is the major ligand of CCR5; however, it also acts as a ligand for CCR1 and CCR3 and influences monocyte and T-lymphocyte migration [98,99]. In vitro studies suggest that at the spinal cord level, CCL5 can be produced by microglial and astroglial cells [56]. The pronociceptive properties of CCL5 have already been suggested since intrathecal injection of CCL5 induced strong mechanical and even stronger thermal hypersensitivity, which lasted until 48 h after chemokine administration [16]. To date, the upregulation of the CCL5 protein level after nerve injury and in bone cancer pain has been shown in rats [26,100] and mice [16]. Moreover, CCL5-knockout mice develop lower hypersensitivity after partial sciatic nerve ligation [101], and CCL5-neutralizing antibodies reduce pain-like behaviors [25,26]. An enhanced CCL5 level is also found in injured nerves, and its blockade by Met-RANTES indicates its potential role in peripheral sensitization [102,103]. Moreover, the intrathecal administration of an anti-CCL5 neutralizing antibody attenuates established hyperalgesia in rats with bone cancer pain [100]. Nevertheless, the relatively small and short-lasting spinal increase in the CCL5 level after nerve injury suggests that its contribution to central sensitization is less important than that of other chemokines, especially CCL2, CCL7, and CCL8.

**CCL6** is the selective ligand of CCR1 produced by monocytes, macrophages, microglia, and eosinophils [104,105]; however, its role in nociceptive transmission is poorly understood. Long-lasting spinal *CCL6* upregulation at the mRNA level was shown after CCI injury in mice [16] and rats [53]. However, in contrast, a slight spinal downregulation of the CCL6 protein level was observed [16], which suggests a less important role for this chemokine in nociception in rodents. Moreover, CCL6 is not present in humans; however, it is considered a human ortholog of **CCL15** and **CCL23 [106]**. Importantly, in the cerebrospinal fluid of patients with neuropathic pain, increased CCL23 levels have also been observed [107]; therefore, it would certainly be worth checking the influence of CCL15 and CCL23 on nociceptive transmission and their involvement in the development of hypersensitivity in neurodegenerative and autoimmune diseases.

**CCL7**, known as a pleiotropic pronociceptive factor, can bind to CCR1, CCR2, CCR3, and CCR5. It was already shown that a single intrathecal injection of CCL7 into naive mice induces strong pain-related behavior, which lasts at least up to 48 h [15,16]. Moreover, after nerve injury, CCL7 may be released by macrophages, neurons, and astrocytes [108,109,110]. Importantly, this chemokine can evoke the activation and chemotaxis of many cells, e.g., microglia, macrophages, and neutrophils [59,111,112], which are known to be important in neuropathy. Moreover, nerve ligation sustainably increases the level of CCL7 in the spinal cord [23,59] and DRGs [23]. Furthermore, CCL7-neutralizing antibodies effectively decrease pain-related symptoms after nerve injury [15] and suppress microglial cell activation [59]. It was also shown that CCL7-knockout mice develop symptoms of neuropathic pain to a lesser extent [108]. Moreover, it was proven that neuron-derived CCL7 promotes glial activation during neuropathic pain [108]. Imai et al. suggested that increased CCL7 expression may serve to ease the interactions between astrocytes and microglia in the spinal cord and could play an essential role in neuropathic pain [59]. Later, in vitro studies revealed that both microglia and astrocytes may release CCL7 [15], indicating that these cells may be activated in a paracrine and autocrine manner by those chemokines. Interestingly, Li et al. reported that decreasing CCL7 levels is more effective for pain relief than decreasing CCL2 levels in a spinal nerve ligation model [113]. This finding agrees well with subsequent behavioral data, which demonstrated that intrathecal injection of a CCL7-neutralizing antibody effectively attenuates CCI-induced neuropathic pain in mice at lower doses than those required for a CCL2-neutralizing antibody [15]. Published data indicate that the long-lasting upregulation of CCL7 induced by nerve injury is associated with enhanced multidirectional spinal communication between neurons, microglia, and astrocytes, which leads to sensory neuron sensitization in the early and late phases of neuropathy.

**CCL8**, which binds to CCR1, CCR2, CCR3, and CCR5, is one of the most elevated chemokines in the spinal cord after CCI from the early to late phases of neuropathic pain [16]. Nevertheless, to the best of our knowledge, there are only a few studies on the role of CCL8 in painful neuropathy. The recent literature indicates that this chemokine is particularly important because CCL8 levels have already been reported to be increased in the CSF of patients with neuropathic pain [107]. It was previously shown that CCL8 injected intrathecally causes mechanical and thermal hypersensitivity with greater potency than other chemokines [16]. In the case of thermal stimulation, pain-related behaviors were observed even 48 h after chemokine administration [16]. Moreover, the secretion of this chemokine by neurons and macrophages has already been documented [114,115]. Based on the abovementioned data, we suggest that CCL8 may be one of the most important chemokines in nociceptive transmission, especially because it is also known that the inhibition of CCL8 may decrease visceral hyperalgesia [114]; however, experimental and clinical studies are still needed to investigate its exact role in neuropathy.

Similar to the aforementioned CCR1 ligands, CCL9 also seems to have an important role in nociceptive transmission. Intrathecal injection of CCL9 evokes mechanical and thermal hypersensitivity in naive mice [16,19]. This result is important, among other reasons, because CCL9 is a selective CCR1 ligand, and its pronociceptive effects clearly confirm the important role of CCR1 in the development of hypersensitivity. It was already shown that in a mouse model of diabetic neuropathic pain, strong spinal upregulation of CCL9 is in close association with the development of hypersensitivity [19] and that its neutralization by antibodies reduces pain-related symptoms [19]. Moreover, although in rats [53]/mice [16], increased levels of *CCL9* mRNA in the spinal cord and/or DRGs are observed at many time points after injury, the protein level of CCL9 is enhanced between the 1st and 7th days post-CCI [53,57] and 7 days after streptozotocin injection [19]. Therefore, based on published results, we believe that CCL9 plays an important role in the early phase of neuropathic pain development. The immunohistochemical results provide evidence that CCL9 colocalizes with the neuronal marker NeuN, indicating that neurons are the main source of this chemokine [19]. Although CCL9 is not expressed in humans, this chemokine (similar to CCL6) has an ortholog, CCL23, which is known to be upregulated in the CSF of patients with neuropathic pain [107]. The literature results indicate that CCL9 (similar to CCL3) plays an important role in the development of neuropathy in rodents, unlike the previously mentioned CCR1 ligands CCL2, CCL7, and CCL8, which are responsible for both the development and maintenance of pain.

In the case of **CCL13**, **CCL14**, and **CCL16**, to our knowledge, there are no data showing their influence on neuropathy in humans or even in animal models (excluding mice and rats, which do not express these chemokines).

**CCR2** is preferentially bound by CCL2 [9]; however, other chemokines that have pronociceptive properties, such as CCL7, CCL8, CCL12, and CCL13, are also able to bind to this receptor. The strong pronociceptive properties and role in neuropathy of CCL2, CCL7, and CCL8 have already been discussed above, as they are also CCR1 ligands. The remaining two chemokines still require thorough research; data on the role of CCL12 are ambiguous, while the possible involvement of CCL13 in nociception processes remains to be investigated.

**CCL12** seems to be a selective ligand of CCR2. In vitro studies have provided evidence that CCL12 can be expressed by microglia and astrocytes [15]. Moreover, it was shown that *CCL12* mRNA was slightly upregulated in the spinal cord 2 days after CCI [15] and then gradually decreased until Day 14 [15]; however, the protein level has not yet been studied. Its enhanced level is also observed in DRGs 7 days after CCI [60]. *CCL12* is also one of the most upregulated genes after pSNL, as measured in FACS-sorted microglia from the spinal cord [116]. Considering the high homology between CCL2 and CCL12 and their similar mRNA changes after nerve injury [15], it would be expected that CCL12 also has strong pronociceptive properties. However, the intrathecal injection of CCL12 does not induce any pain-related behaviors in naive animals [15]. Therefore, in light of published data, it seems that spinal CCL12, in contrast to other CCR2 ligands, is not crucial for the development of pain-related behavior. To date, the upregulation of CCL12 has been demonstrated in the articular cartilage of osteoarthritic knees; therefore, its participation in joint pain has been proposed [116,117]. However, there is still a lack of data explaining the actual role of CCL12 in the CNS, which is why further studies in different animal models are needed.

**CCR3** also seems to be an important receptor for nociceptive transmission since it is a target of several chemokines, including **CCL5**, **CCL7**, **CCL8**, **CCL11**, **CCL13**, **CCL15**, **CCL24**, **CCL26**, and **CCL28.** The strong pronociceptive properties of three of them, namely, CCL5, CCL7, and CCL8, have been described above; however, it remains unclear which of the chemokine receptors is most involved in their pleiotropic pronociceptive effects. Importantly, the role of other ligands of this receptor, which have been studied to a lesser extent thus far, remains to be clarified.

**CCL11** also seems to be significant for nociceptive transmission; it has a very high affinity for CCR3 and has been shown to play a crucial role in the recruitment of eosinophils, basophils, neutrophils, and macrophages [118,119]. In the CNS, CCL11 is secreted by microglia and astrocytes [120,121]. In CCI-induced neuropathic pain, the long-term increase in *CCL11* mRNA in the spinal cord and/or DRGs is known to occur in rats [23] but not in mice [16]. Nevertheless, 7 days after CCI, enhanced protein levels of CCL11 are observed in DRGs [23]. Notably, a high level of this chemokine has also been observed in the CSF of patients with neuropathic pain, indicating its significant role in neuropathy [107]. Moreover, CCL11 is currently suggested to be a biomarker of fibromyalgia [122]. In addition, it has been shown to be involved in pain development in osteoarthrosis [123]. Published data indicate that CCL11 acts via CCR3, CCR5, and CXCR3; therefore, CCL11 should be considered a potential target for pharmacotherapy. In our opinion, it would undoubtedly be worth using an anti-CCL11 monoclonal antibody in experimental studies to prove this hypothesis. In particular, recently, bertilimumab, a humanized monoclonal antibody against CCL11, has been used in clinical trials for treating severe allergic disorders, skin disorders, and inflammatory bowel disease [124,125].

Unlike the already-discussed **CCR3** ligands, the other three CCLs, **CCL24**, **CCL26**, and **CCL28**, are still poorly studied. Experimental studies have shown only that after CCI, the spinal mRNA levels of *CCL24* [16] and *CCL28* [16] are unchanged in mice, and *CCL28* is undetectable in rats [23]. Moreover, *CCL26* levels are slightly elevated, but only in the late phase—on Day 28 in rats—while there is no detection of this chemokine in the spinal cords of mice [16,23]. Interestingly, a recent clinical study provided evidence that the serum level of CCL24 is higher in patients with fibromyalgia [126], which suggests its important role in peripheral sensitization. The available literature indicates that CCR3 and its ligands are potential targets for pain relief; however, more studies are needed.

Published data indicate that **CCR4** has two selective ligands, **CCL17** and **CCL22 [127,128]**, and one nonselective ligand, **CCL2**. CCL17 is known to be secreted by lymphocytes, monocytes, dendritic cells, and neurons [129], while CCL22 is secreted by macrophages, dendritic cells, B and T lymphocytes, and monocytes [129,130]. Importantly, after nerve injury, the spinal levels of *CCL17* and *CCL22* mRNA remain unchanged, unlike the level of *CCL2*, which is highly upregulated [80]. Pharmacological studies have proven that intrathecally administered CCL2 evokes pain-related behavior in naive mice through CCR4 [80]. Subsequent results show that in the peripheral nervous system (PNS), the situation is different, and all three CCR4 ligands may be involved in nociceptive transmission. It has been shown that after CCI, the DRG levels of *CCL17*, *CCL22*, and *CCL2* are strongly upregulated in rats [27,53]. It was also revealed that CCL17 levels are increased in patients suffering from fibromyalgia [122] and diabetic retinopathy [131]. Moreover, the importance of CCL17 and CCL22 during tissue repair in diabetes was recently proven [132]. Based on published data, we believe that in nociceptive transmission at the CNS and PNS levels, the CCL2/CCR4 and CCL2/CCL17/CCL22/CCR4 axes are involved, respectively.

**CCR5** also seems to be an important receptor for nociceptive transmission because it is a target of several chemokines, **CCL3**, **CCL4**, **CCL5**, **CCL7**, **CCL8**, **CCL11**, and **CCL13**, all of which, except for CCL13, as described above, have strong pronociceptive properties, and most of them are implicated in the development and/or maintenance of neuropathy. CCL13 is not present in mice/rats; however, recently, in patients, it was identified as a potential biomarker for abdominal pain in irritable bowel syndrome [133] and osteoarthritis [134]; therefore, its role in neuropathic pain needs to be studied.

**CCR6** is a target of **CCL20**; however, there are few data in the literature suggesting its role in neuropathy; therefore, further studies are necessary. This chemokine is expressed by the endothelium, macrophages, and Th17 lymphocytes [135]. In 2022, it was reported that in patients who developed persistent postoperative neuropathic pain (PPSNP) after breast cancer surgery, the level of CCL20 was increased [136]. Moreover, other recently published data have shown that a high level of CCL20 is strongly associated with thermal allodynia in patients after traumatic nerve injuries [137].

**CCR7** is a target of two chemokines, **CCL19** and **CCL21**. In light of the available literature, it remains unclear whether the CCL19/CCR7 and CCL21/CCR7 axes play a role in central sensitization. CCL19 may be secreted by mature dendritic cells [138]. It was already shown that the level of CCL19 increased in the plasma and cerebrospinal fluid of patients with neuropathic pain [107,139]. Furthermore, it was reported that CCL19 plays a crucial role in hypersensitivity, which develops during orofacial pain [140]. Undoubtedly, CCL21 plays an important role in central sensitization; however, despite belonging to the CC group, CCL21 shows an affinity for CXCR3-expressing microglia [141,142]. Published data provide evidence that the CCL21/CXCR3 axis is extremely important in the development of neuropathic pain symptoms [143], which will be discussed in the next section.

**CCR8** in rodents is preferentially bound by **CCL1**, which is secreted by various cells, such as neurons, lymphocytes, monocytes, mast cells, epithelial cells, and endothelial cells [13,144]. Recently, it has been shown that recombinant CCL1 injected intrathecally into naive mice induces hypersensitivity [13,14] and, in parallel, activates glia to express higher levels of pronociceptive factors, such as interleukins (ILs): *IL-1beta* and *IL-6* [14]. Moreover, CCL1/CCR8 signaling is suggested to be important for the development of neuropathic pain, since the spinal upregulation of CCL1 was demonstrated in STZ-, CCI- and PSNL-induced neuropathy [13,14,145]. Additionally, nerve-injury-induced hypersensitivity is reduced by CCL1-neutralizing antibody administration [13,14] and in CCR8-knockout mice [14]. Furthermore, current evidence shows that CCL1 is mainly produced in DRG neurons but secreted in the dorsal horn of the spinal cord [14]. It was proven that CCL1 injection evoked the activation of glial cells and the upregulation of proinflammatory cytokines [14]. Immunofluorescence staining proved that CCL1 colocalized with the neuronal marker NeuN [13]. Another ligand that binds to CCR8 is **CCL18**; however, it is not present in mice and rats, and thus far, its role in neuropathy in patients needs to be studied. Experimental data provide valuable insight into the CCL1/CCR8 signaling pathway as a novel therapeutic target for neuropathic pain.

**CCR9** is a selective target for **CCL25**. The role of the CCL25/CCR9 axis in the development of neuropathy requires research; however, in 2022, it was shown that in the CSF of patients who developed postoperative neuropathic pain after breast cancer surgery, an enhanced level of CCL25 was observed [136].

**CCR10** is a target for three chemokines, **CCL26**, **CCL27**, and **CCL28**. To date, none of these chemokines have been proven to play an essential role in nociceptive transmission, and some clinical studies are needed.

In summary, the literature provides evidence that many endogenous ligands of chemokine receptors, such as CCL1/2/3/5/7/8/9/11, are significant in the development (CCL2/3/5/7/8/9) and maintenance (CCL2/7/8) of neuropathic pain. Among them, CCL2/7/8 seem to be the most important because of the quick and long-lasting increase in their protein levels and strong pronociceptive properties. Importantly, it was shown that repeated injections of bindarit, an inhibitor of CCL2/7/8 synthesis, effectively attenuate pain-related behaviors in different phases of neuropathic pain development in mice [16]. Bindarit has analgesic effects even in fully developed neuropathy. The obtained results provide evidence that CCL2/7/8 may serve as potential therapeutic targets for neuropathic pain treatment, regardless of sex [16]. Moreover, in mice, bindarit suppresses cancer pain development [146] and autoimmune encephalomyelitis [147]. Importantly, bindarit was in the second phase of clinical trials for type 2 diabetic nephropathy (NCT01109212) [146]. The available literature clearly indicates that in the CNS, the CCL2/3/5/7/8/9/CCR1, CCL2/7/8/CCR2, CCL5/7/8/11/CCR3, CCL2/CCR4, CCL3/5/7/8/11/CCR5, and CCL1/CCR8 axes and, in the PNS, the CCL17/22/CCR4 axes are especially important in the development and/or maintenance of neuropathic pain. The results indicate that CC family members are promising targets in the search for neuropathic pain therapy; however, more studies are needed.

### 1.2. CXC Chemokines in Neuropathic Pain

The CXC subfamily is the second-largest group of chemokines in terms of the number of members (consisting of 17 members) (Table 1) and is characterized by a single amino acid between the first two cysteine residues. Neuropathic pain caused by sciatic nerve damage has also been shown to be associated with strong time-dependent changes in the levels of many endogenous chemokine receptor ligands, including CXCR2-CXCL1, CXCL2, and CXCL3 [31]; CXCR3-CXCL4, CXCL9, CXCL10, and CXCL11 [28]; and CXCR1/CXCR2-CXCL5 [34]. In addition, 10 chemokines acting through CXC receptors have a strong pronociceptive effect, namely, CXCL1 [31]; CXCL2 [31]; CXCL3 [31]; CXCL4 [28]; CXCL5 [34]; CXCL9 [28]; CXCL10 [28]; CXCL11 [28], CXCL17 [42], and CCL21 [28].

The role of CXCR1 in the processes of nociception remains unclear; however, recently, it was proven that this receptor is expressed by spinal cord neurons and upregulated during the development of bone cancer pain [148]. CXCR1 has four ligands: CXCL5, CXCL6, CXCL7, and CXCL8. The intrathecal administration of CXCL5 induces nociceptive hypersensitivity in naive rats [34]. Moreover, CCI-evoked pain is accompanied by an increase in spinal CXCL5; however, the involvement of CXCR1 in this phenomenon remains unclear since this chemokine also acts via CXCR2. Studies have also shown its protein upregulation in a diabetic neuropathy model [62]. Importantly, the intrathecal administration of a CXCL5-neutralizing antibody diminished neuropathic-pain-related behavior [34]. Importantly, an increased level of CXCL5 is also observed in the CSF of patients with neuropathy, similar to the level of CXCL6 [107], but in rats, after CCI, the CSF level of CXCL6 is not changed [64]. However, whether CXCL6 and CXCL7 are involved in nociceptive transmission still needs to be investigated. The most extensively studied ligand of CXCR1 is CXCL8, previously known as IL-8, which is also the ligand of CXCR2. This chemokine is produced by neutrophils, macrophages, T lymphocytes, endothelial cells, and epithelial cells [149]. The CXCL8 level increases in the injured sciatic nerve shortly after PSL [66]. In patients suffering from polyneuropathies, the level of CXCL8 was also enhanced in peripheral blood mononuclear cells [150] and in the serum of those with diabetic neuropathic pain [151].

**CXCR2** seems to be one of the most significant CXC receptors involved in neuropathy. CXCR2 acts as a pronociceptive ligand belonging to the subfamily called cytokine-induced neutrophil chemoattractants (CINCs), which are closely related to each other; however, they have different tissue expression and regulation. The CINC family arose as a result of two rounds of gene duplication in the course of evolution [152] and currently includes CINC-1 (**CXCL1**), CINC-2 (**CXCL3**), and CINC-3 (**CXCL2**). CXCL1 has the highest efficacy against CXCR2 and intermediate efficacy against CXCL2 and CXCL3; however, all are expressed by macrophages and play important roles in neutrophil infiltration [153,154]. Although the intrathecal administration of CXCL1 and CXCL2 evoke mechanical and thermal hypersensitivity in naive mice [31], their spinal protein levels remain unchanged after a chronic constriction injury of the sciatic nerve [31]. In contrast, CXCL1 is highly upregulated in the spinal cord and/or DRGs following spinal nerve ligation [32,155]. Both chemokines are increased in a diabetic neuropathy model [62,63], but with CXCL1 in the spinal cord and CXCL2 in the sciatic nerve. Interestingly, the neutralization of spinal IL-17 alleviates neuropathic pain evoked by CCI by reducing the CXCL1 level [156]. Others have also shown that CXCL2 levels are enhanced in infiltrating neutrophils and macrophages in an injured sciatic nerve after its ligation [33]. Based on published data, we hypothesize that the CXCL1/CXCR2 and CXCL2/CXCR2 axes play an important role in nociception; however, their impact seems to be more significant in the PNS than in the CNS. Importantly, the latest data indicate that among CINCs, CXCL3 seems to play a crucial role in the CNS since it is highly upregulated at the spinal cord level after nerve injury [31]. Research on this chemokine has only recently begun, as its biophysical/structural characteristics were previously unavailable [152]. Recent studies have shown that although the overall oligomerization features of all CINCs are similar, prominent differences can be observed in their characteristic surface structures, thus indicating functional divergence [152]. CXCL3 exerts its effects via CXCR2 through signaling pathways, such as p38MAPK and ERK1/2 [157,158], which are known to be important factors in neuropathy [159]. Moreover, the behavioral results provide evidence for the pronociceptive properties of CXCL3, where, after its intrathecal administration, hypersensitivity appears quickly, which is connected to the fact that CXCR2 is present on neuronal cells [31]. Moreover, the CXCL3-neutralizing antibody diminishes pain-related behavior evoked by nerve injury [31]. Additionally, CXCL3 is responsible for neutrophil recruitment and acts as a mediator of macrophage chemotaxis, which is important since these cells are crucial in neuropathic pain pathogenesis [160]. The abovementioned results indicate the important role of CXCL3 in both the initiation and maintenance of neuropathic pain; therefore, in our opinion, both CXCL3 secretion and CXCR2 blockade can have beneficial effects, which may help relieve the symptoms of neuropathic pain.

The ligands of **CXCR3** (**CXCL4**, **CXCL9**, **CXCL10**, **CXCL11**, and **CCL21**) may also evoke strong pain-like behaviors in naive mice after a single intrathecal administration [28]. Importantly, different time course changes are observed after nerve injury [28,30]. On Day 2 after injury, at the spinal cord level, increases in CXCL10 and CXCL11 were observed, indicating their role in triggering neuropathy [28]. Then, on Day 7, increases in CXCL4, CXCL9, and CXCL10 were also measured. However, long-lasting changes (until Day 28) were observed only for CXCL9, suggesting that this chemokine is responsible for persistent neuropathy [28]. Additionally, in DRGs, CXCL4 and CXCL9 levels were elevated 7–28 days after injury, which can be important for peripheral sensitivity development [28].

Changes in the levels of **CXCL4** observed in the CNS and PNS indicate the important role of this chemokine in nociceptive transmission [28]. The in vivo and in vitro results are particularly interesting, as they indicate that CXCL4 is specifically expressed by microglia but not by astroglia or neurons [161]. It was also proven that microglial migration induced by CXCL4 is absent in CXCR3-deficient microglia [161]. The exact role of this chemokine in neuropathy, especially in DRGs, should undoubtedly be the subject of further research.

However, among the tested CXCR3 ligands, the role of **CXCL9** seems to be the most important due to its strong pronociceptive effects [28] and long-lasting upregulation after nerve injury observed at the spinal cord and DRG levels [28,67]. Moreover, the spinal upregulation of CXCL9 has already been described in diabetic neuropathy [62] and in locally inflamed DRGs [68]. Published data indicate that CXCL9 is expressed by neurons, microglia, and astroglia [28,162]. Moreover, the administration of a CXCL9-neutralizing antibody diminishes the pain-related behavior observed after nerve injury [28]. Therefore, CXCL9 appears to be a very important nociceptive mediator in neuropathy and is particularly crucial for the persistence of neuropathy.

The role of **CXCL10** in nociceptive transmission seems to be very important since numerous studies have described its role in this process: its spinal upregulation has already been proven in many animal models, e.g., SNL-, CCI-, CIBP-, and STZ-evoked neuropathy [28,35,36,68,163,164]. Moreover, it was already shown that the intrathecal administration of a CXCL10-neutralizing antibody reduces the development of CCI-evoked neuropathic pain [28] and cancer-induced bone pain [36]. It is known that at the spinal cord level, CXCL10 enhances the amplitude of spontaneous excitatory postsynaptic current and increases NMDA-/AMPA-induced currents via CXCR3; these results support a role for the CXCL10/CXCR3 axis in facilitating excitatory synaptic transmission in neuropathy [163]. Moreover, in the SNL model, increases in *CXCL10* mRNA and the number of action potentials evoked by this chemokine were observed in DRGs [67]. Notably, the intrathecal administration of CXCL10 produces rapid and CXCR3-dependent pain hypersensitivity [28,163]. CXCL10 may also induce AKT and ERK activation in trigeminal ganglion neurons and contribute to the maintenance of neuropathic pain [165]. Interestingly, the levels of CXCL10 in blood samples were elevated in patients with diabetic polyneuropathy compared to patients without diabetes [166]. Therefore, in our opinion, the modulation of CXCR3/CXCL10 signaling can bring satisfactory pain relief in neuropathy.

Published data suggest that **CXCL11** plays an important role in the first phase of the development of pain [28,167]. Intrathecal administration induces quick but short-term hypersensitivity in naive mice [28]. The upregulation of its mRNA is also observed after SNL [67] and in a diabetic model of neuropathy [62], while its protein level is slightly upregulated after CCI [28]. Moreover, in vitro results suggest that CXCL11 can be produced by microglial and astroglial cells [28,168]. However, based on available results, it is not possible to determine the exact role of CXCL11, and this issue requires future study.

Importantly, **CCL21**, which also binds to CXCR3, similarly has strong pronociceptive properties. It was already proven that this chemokine is not detected under physiological conditions in the CNS, but after injury, it is strongly upregulated [30,61,169]. Its increase is also observed after the use of anticancer drugs [29]. In neuropathy, CCL21 is expressed in the DRGs in injured small-diameter primary sensory neurons [30,170] and is immediately transported to axons within the dorsal horn of the spinal cord [161,171]. At the spinal cord level, CCL21 evoked strong microglial cell activation [141]. Therefore, the administration of a CCL21-neutralizing antibody attenuates the development of hypersensitivity in neuropathic pain models [28,30]. It has also been proven that in CCL21-knockout mice, pain-related behavior develops to a lesser extent after nerve injury [143]. Therefore, based on the literature, we think that CCL21-CXCR3 signaling is also strongly involved in the development and maintenance of neuropathic pain.

The involvement of **CXCR4/CXCL12** in pathological neuropathic pain has been broadly studied [39]. Nerve injury evokes an increase in CXCL12 in DRGs [38,69] and in the spinal cord [39], suggesting that this chemokine may participate in hypersensitivity development both peripherally and centrally. Similarly, streptozotocin-evoked diabetic neuropathy leads to its protein upregulation in the spine [62] and in the area of the anterior cingulate cortex [70]. CXCL12/CXCR4 signaling is involved in pain development in a model of bone cancer [172] and SNL- [37], CCI- [173], and SNI-induced [38] neuropathy by sensitizing neurons or activating astrocytes and microglia. Moreover, it was proven that the intrathecal injection of CXCL12 affects the development of hypersensitivity in naive rats [39], while the intrathecal injection of a CXCL12-neutralizing antibody diminishes neuropathic pain development and maintenance [39]. In light of the obtained results, the authors agree that CXCL12/CXCR4 signaling may serve as a novel target that can be exploited for the treatment of neuropathic pain.

CXCR4 has a positive allosteric modulator, CXCL14 [174], that is constitutively expressed in the CNS [175] and PNS [176]. First, a microarray analysis of rat DRGs showed that local inflammation evokes the significant upregulation of CXCL14 in parallel with the development of hypersensitivity [68]. Later, increased spinal CXCL14 was described as involved in the development of paclitaxel-induced [76] neuropathic pain. Moreover, the use of CXCL14 siRNAs significantly attenuates hypersensitivity induced by paclitaxel [76]. Recently, it was proven that CXCL14 contributes to the modulation of hypersensitivity, together with somatostatin [177]. CXCL14 is currently suggested to be a crucial factor in the initial phase and maintenance of neuropathic pain.

**CXCR5** is a selective target of **CXCL13**; however, under physiological conditions in the CNS, it is expressed at a very low level [40]. Nevertheless, CXCL13 mRNA and/or protein levels increase in the spinal cord and/or DRGs in SNL- [40,68,72], CCI- [75], SpNI- [73], DB- [71], and TNFα-induced [74] neuropathic pain models. The intrathecal injection of CXCL13 induces pain hypersensitivity and astrocyte activation via CXCR5 [40]. Moreover, CXCL13 promotes the production of cytokines to elicit hypersensitivity [71]. The shRNA-mediated spinal inhibition of CXCL13 diminishes SNL-induced neuropathic pain [40], and the spinal overexpression of miR-186-5p is able to reduce CXCL13 expression [40]. Moreover, the DRG microinjection of *CXCL13* siRNA reduces SNL-induced hypersensitivities [72], while the neutralizing antibody diminishes CPIP-evoked neuropathic pain symptoms [41]. Moreover, it was shown that the neuronally enhanced production of CXCL13 after nerve injury contributes to neuropathic pain development through ERK-mediated nociceptive factor release [178,179]. Therefore, in our opinion, CXCL13 seems to also be a key player in neuropathic pain pathogenesis.

**CXCR6** is a selective target of **CXCL16**; however, whether this chemokine is involved in nociceptive transmission still needs to be investigated. CXCL16 is composed of a mucin-like stalk, a transmembrane domain, and a cytoplasmic tail containing a potential tyrosine phosphorylation site that may bind to SH2 [180]. These are unusual features of CXCL16 and allow it to be expressed as a soluble form and a cell-surface-bound molecule [181]. Moreover, the expression of CXCL16 is induced by the inflammatory cytokines IFN-gamma and TNF-alpha [181]. The involvement of this chemokine in central sensitization has not been demonstrated, although, to the best of our knowledge, there are two papers that show, first, its changes after spinal cord stimulation [182] and, second, the slight upregulation of its gene 2 weeks after CCI [183]. More studies are needed.

**CXCR8** is a target of CXCL17 [184,185]. This chemokine was one of the latest to be identified, that is, in 2006 [186]. To date, only one study has shown that the intrathecal administration of CXCL17 in naive mice induced strong tactile and thermal hypersensitivity [42]. Moreover, the author has shown that the effect is abolished by kynurenic acid and zaprinast administration [42]. The observed pronociceptive properties of CXCL17, which is a strong monocyte chemoattractant [184,187], are very important and may play a role in the development of neuropathy. Transcriptomic analysis has shown that *CXCL17* is elevated after CCI in the anterior cingulate cortex and spinal cord [183]. However, more studies are needed since some studies have shown that CXCL17 has anti-inflammatory effects on LPS-activated macrophages by suppressing the production of proinflammatory cytokines [187]. Notably, it was recently shown that CXCL17 is a promising therapeutic target and is an independent biomarker of poor prognosis in patients with breast cancer [188]. The data in the literature need to be completed since those that already exist indicate that the modulation of the CXCL17/CXCR8 axis may become a potential strategy for the treatment of pain.

**CXCL15** remains poorly understood in the context of nociception processes, and its receptor remains unknown. Moreover, CXCL15 is a typical murine chemokine that is not found in humans, and importantly, CXCL15 is mainly expressed in the lungs, digestive tract, and urogenital organs [189]; therefore, its role in nociceptive transmission seems unlikely.

In sum, the abovementioned results indicate that many endogenous ligands of chemokine receptors of the CXC subfamily are very important for nociceptive transmission. It seems that in the CNS, the axes CXCL3/CXCR2, CXCL9/CXCR3, CXCL10/CXCR3, CXCL12/CXCR4, CXCL13/CXCR5, CXCL14/CXCR4, CXCL17/CXCR8, and CCL21/CXCR3 and, in the PNS, the axes CXCL1/CXCR2 and CXCL2/CXCR2 are especially crucial during the development of neuropathic pain. The results indicate that these chemokines and their receptors may be promising targets in the search for pharmacotherapeutic agents to treat neuropathic pain; however, more studies are needed.

### 1.3. XC Chemokines in Neuropathic Pain

The XC subfamily consists of two members, **XCL1** and **XCL2**, both of which are present in humans, but there is only XCL1 in the mouse and rat genome [190]. It was shown that both of these closely related chemokines act through XCR [191]. XCL1 is produced by immune cells and astrocytes [43,192]. First, in 2016, it was found that at the spinal cord level, XCL1 is highly upregulated in diabetic neuropathy [44]; later, in 2022, its time-dependent and long-lasting changes after sciatic nerve injury were described [43]. Moreover, in this study, using immunofluorescence, the authors showed that XCL1 is released at the spinal cord level, mainly by astroglial cells, but XCR1 is present mostly on neuronal cells [43]. Furthermore, it was proven that the intrathecal administration of XCL1 evokes hypersensitivity in naive mice [43,44]. Importantly, XCL1-neutralizing antibody administration attenuates hypersensitivity development in STZ- and CCI-evoked neuropathic pain in mice [43,44]. Recent research shows that XCL1 affects fibroblast migration through an atypical chemokine receptor, the heterodimeric (αβ) transmembrane receptor ITGA9 [193]. Pharmacological studies published in 2022 gave the first evidence that the blockade/neutralization of both receptors, XCR1 and ITGA9, reverses hypersensitivity evoked by intrathecal XCL1 administration in naive mice; however, the neutralization of ITGA9 is more effective. These data clearly indicate that XCL1 exhibits strong pronociceptive properties not only through XCR1 but also through ITGA9, which is also localized on neurons [43]. So far, only changes in the level of XCL2 have been studied in patients with neuropathy, and its downregulation has been observed; however, the authors indicated that more research is needed because this observation may be related to drug treatment [194]. Importantly, the neutralization of XCL1 improves morphine analgesia [43]. Moreover, the blockade of XCR1 positively influences buprenorphine effectiveness, and the neutralization of ITGA9 enhances not only buprenorphine but also morphine analgesia [43]. In summary, experimental studies suggest that both the XCL1/XCR1 and XCL1/ITGA9 axes play a role in neuropathic pain development; however, ITGA9 seems to be a more important neuronal target [43]. Therefore, it seems that the blockade of the XCL1/ITGA9 axis may serve as an innovative strategy for the polypharmacotherapy of neuropathic pain in combination with opioids [43]. Nevertheless, the role of XCL1 and XCL2 in neuropathic pain in humans needs to be elucidated.

### 1.4. CX3C Chemokine in Neuropathic Pain

Only one chemokine belongs to the CX3C subfamily, namely, **CX3CL1**, which occurs in two forms, membrane-bound or soluble [195]. CX3CL1 (also known as fractalkine) was one of the first described to be important for nociceptive transmission. It was shown that intrathecal CX3CL1 administration evokes strong mechanical and thermal hypersensitivity [45]. It was also shown that the mRNA level of this chemokine is elevated in the spinal cords of animals with neuropathy evoked by TNF-α injection [74]. Additionally, its upregulation is observed in the oxaliplatin model of neuropathic pain [46]. CX3CL1-induced pain hypersensitivity is abrogated in CX3CR1-knockout mice [196]. Moreover, neutralization of the chemokine diminishes pain-like behavior [46]. This chemokine is produced by neurons in the spinal cord and DRGs after nerve injury, while its receptor, CX3CR1, is present on the surface of microglial cells and is highly upregulated during neuropathic pain development [8]. It is also known that the binding of CX3CR1 by CX3CL1 increases microglial proliferation and migration [197,198]. There is also evidence that ERK5, which is expressed by microglia, is necessary for CX3CL1/CX3CR1-induced microglial activation and the induction of hyperalgesia [199]. CX3CR1 activation by CX3CL1 causes the phosphorylation of p38 MAPK, which results in the production of many pronociceptive factors, including TNFα, IL-1β, and IL-6 [9]. The link between CX3CL1/CX3CR1 signaling, microglial phenotypes, and neuronal damage/loss has long been proposed, with a growing body of data on the possible role of the CX3CL1/CX3CR1 axis in neurodegeneration. Molecules that target CX3CL1 may provide reduced side effects and stronger analgesia [200]. In sum, it seems that the modulation of the CX3CL1/CX3CR1 axis may bring some new benefits to neuropathic pain therapy. However, there is still a lack of selective safe substances that are able to cross the blood–brain barrier.

## 2. Chemokine Receptors and Neuropathic Pain

As mentioned in the previous section, chemokines activate specific receptors located on the surface of various immune, glial, and neural cells; twenty receptors have been characterized thus far [8,201]. Each is a seven-transmembrane G-protein-coupled receptor (Gαi) [8]. Published data indicate that the pharmacological blockade of some chemokine receptors from the CC group, as well as from the CXC, XC, and CX3C groups, relieves neuropathic pain of various etiologies in mice and/or rats, which we will discuss in detail in the following sections.

### 2.1. Analgesic Potential of Targeting Single CC Chemokine Receptors

Published data indicate that the blockade of five CC receptors causes analgesia and improves the effectiveness of opioids in different neuropathic pain models (Table 3).

#### 2.1.1. CCR1

The involvement of CCR1 in the development of hyperalgesia is still insufficiently investigated. However, it was recently shown that single and repeated intrathecal administration of the CCR1 antagonist J113863 significantly attenuates mechanical and thermal hypersensitivity in STZ [19], CCI [16,53], and cancer pain [207] models. Furthermore, the blockade of CCR1 by BI64 reduced hypersensitivity in a rat inflammatory pain model [208]. These beneficial analgesic effects of antagonists are undoubtedly related to both the cellular localization of CCR1 and spatiotemporal changes in its ligands. CCR1 is localized on neuronal, glial (astrocytes, microglia), and immune (neutrophils, basophils, monocytes, eosinophils, lymphocytes) cells [120,209,210,211,212,213]. CCR1 seems to be a very important receptor for nociceptive transmission since it has several ligands with strong pronociceptive properties, such as CCL2, CCL3, CCL4, CCL5, CCL7, CCL8, and CCL9 [9,15,19,214,215,216,217,218,219]. It was already shown that in the early phase of neuropathic pain development, increases in CCL2, CCL3, CCL, CCL5, CCL7, CCL8, and CCL9 are observed, and in the maintenance phase, CCL2, CCL7, and CCL8 are observed [16]. Moreover, the neutralization of spinal CCL2, CCL3, CCL7, and CCL9 by their antibodies strongly attenuated neuropathic pain symptoms [15,19]. However, to date, it is still not known how and whether CCR1 blockade affects the biosynthesis of pronociceptive chemokines. Evidence shows that CCR1 is upregulated after nerve injury [220]. Importantly, CCR1 knockdown diminishes nerve pain in rats subjected to SNL and represses microglial activation [221]. Recent research indicates that the repeated intrathecal administration of J113863 reduces the activation and/or infiltration of microglia, macrophages, neutrophils, and lymphocytes into the spinal cord and/or DRGs and thus induces beneficial changes in the levels of factors with pronociceptive (IL-1 beta, IL-6, and IL-18) and antinociceptive (IL-1 receptor antagonist) properties [53]. Moreover, the intrathecal administration of a CCR1 antagonist not only relieves pain-related behavior but also improves the analgesic properties of opioids in STZ [19] and CCI [53] models. The authors suggest that CCR1 blockade probably restores the immune balance, which is one of the main proposed mechanisms by which it improves the effectiveness of opioids in the CCI model [53]. It is currently believed that the low effectiveness of opioid drugs in neuropathy is due to the strong production of pronociceptive factors with anti-opioid properties [222,223,224,225]. Recent studies have proven that a single administration of CCL2-, CCL3-, CCL7-, and CCL9-neutralizing antibodies can intensify morphine and/or buprenorphine analgesia [15,19,219]. The other reason for improved opioid analgesia might be the ability to form dimers between chemokine and opioid receptors, which was already shown for CCR5-MOR [226]. However, there is a lack of data showing that CCR1 can create heterodimers with opioid receptors, but it was shown that CCR1 is able to create dimers with CCR5 [227,228]. The available experimental studies indicate that CCR1 is involved in the pathogenesis of diseases with large neuroimmunological components, such as rheumatoid arthritis [208,210,229], disc inflammation [230], multiple sclerosis [231,232], and diabetes [19]. Importantly, several CCR1 antagonists have entered clinical trials, including MLN3897 and CP-481,715 for rheumatoid arthritis, BX471 for multiple sclerosis, AZD-4818 for chronic obstructive pulmonary disease [233], and BAY86-5047 for endometriosis [234]. In summary, based on available results from experimental and clinical studies, it can be hypothesized that pharmacological modulation via CCR1 may represent a novel strategy for effective polytherapy with opioids in patients suffering from neuropathic pain.

#### 2.1.2. CCR2

Published data indicate that the repeated intrathecal administration of the single CCR2 antagonist RS504393 [52,60] reduces tactile and thermal hypersensitivity in neuropathic pain induced by sciatic nerve ligation in mice and/or rats. Moreover, RS504393 not only attenuates pain-related behavior but also enhances the analgesic properties of morphine and buprenorphine after CCI [52]. Similarly, intracisternal injections of the mentioned CCR2 antagonist reduce neuropathic pain symptoms induced by inferior alveolar nerve transection [204], mental nerve transection [204], and lumbar disc herniation [235]. Moreover, the subcutaneous (s.c.) or intrathecal administration of RS504393 diminishes paclitaxel-evoked pain [203]. Furthermore, the oral administration of another CCR2 antagonist, AZ889, may reverse mechanical and thermal hyperalgesia in CCI-subjected animals [205]. CCR2 is expressed by spinal microglia, astrocytes, and neurons [17,236,237], and it is already known that CCR2 is mainly bound by CCL2 [17]; however, not only CCL2 but also two of its ligands, CCL7 and CCL8, have strong pronociceptive properties. Many studies have shown that these ligands are mediators of spinal glial activation after nerve injury [8,9,51]. Interestingly, the abolition of the development of mechanical hypersensitivity after nerve injury is observed in CCR2-knockout mice [238]. It was already shown that RS504393 diminishes CCI-induced microglial activation at the spinal cord level after CCI [202] and, in parallel, prevents the upregulation of pronociceptive factors, such as IL-1beta, IL-18, IL-6, and inducible nitric oxide synthase, and increases the expression of antinociceptive IL-1alpha [52]. Moreover, it was demonstrated that repeated intrathecal administration of a single CCR2 antagonist (RS504393) significantly reduces the enhanced expression of CCL2, CCL3, CCL4, CCL5, CCL7, and CCL11 in DRGs but does not influence them at the spinal cord level [60]. However, a single intraperitoneal injection of RS504393 does not influence fully developed neuropathic-pain-related behaviors in mice [60]; therefore, further research is needed, which is now enabled by newly synthesized CCR2 antagonists (e.g., INCB 3284, RS 102895, MK-0812, PF-4136309). Additionally, BMS-741672 is in the second phase of clinical trials for patients with diabetic neuropathic pain; however, no study results have yet been posted [239]. Targeting CCR2 through siRNA, blocking antibodies, or small-molecule antagonists may provide new therapeutic possibilities for managing neuropathic pain [240]. The available literature suggests that the pharmacological modulation of spinal neuroimmunological interactions via CCR2 may represent a new strategy for effective polytherapy with opioids in patients suffering from neuropathic pain; however, better pharmacological tools are needed.

#### 2.1.3. CCR3

To our knowledge, the role of CCR3 in nociceptive transmission has not been thoroughly investigated, and the first data were published in 2021. Its presence has been demonstrated in many types of pain-modulating cells, including neurons, microglia, astrocytes, neutrophils, lymphocytes, and satellite cells [23,241,242,243,244,245,246]. Moreover, data in the literature indicate the important role of CCR3 in disorders such as cancer, asthma, atopic skin inflammation, narcolepsy, and inflammatory bone resorption [247,248,249,250,251] and, recently, in neuropathic pain models [16,23]. It was shown that partial infraorbital nerve transection (pIONT) increases the expression of CCR3 [252]. The receptor has several endogenous ligands, such as CCL5, CCL7, CCL8, CCL11, CCL24, CCL26, and CCL28, and importantly, four of them, CCL5, CCL7, CCL8, and CCL11, appear to be important factors involved in nociception processes [15,16,26,56,97,102,107,108,251]. In addition, significant increases in CCL7, CCL5, CCL8, and CCL11 at the level of the spinal cord and/or DRGs have already been demonstrated in the CCI model in mice and rats [16,23]. It was also shown that the single or repeated intrathecal administration of the CCR3 antagonist SB328437 diminishes mechanical and thermal hypersensitivity [23]. Moreover, in parallel, it reduces the CCI-evoked increases in the levels of neutrophil, lymphocyte, and satellite cell protein markers in the spinal cord and/or DRGs, which are accompanied by the downregulation of IL-6, CCL7, and CCL11 [23]. Additionally, the repeated intrathecal administration of the CCR3 antagonist SB328437 enhances the analgesic properties of morphine and buprenorphine, although to a lesser extent for buprenorphine [23]. The mechanism by which SB328437 modulates opioid analgesia remains unclear. One of the reasons could be the heterologous desensitization of CCR3-MOR, as was shown for CCR5-MOR [253,254,255]; however, there are no data showing whether these dimers exist. Another explanation could be that SB328437, through its effects on immune and satellite cells, may participate in the potentiation of opioid analgesia; however, more studies are needed to explore the full mechanism. SB328437 also relieves mechanical hypersensitivity developed in the pIONT model of neuropathy and decreases upregulated pERK levels [252]. A few animal studies have already shown that SB328437 has beneficial effects on carcinoma [256], allergic inflammation [257], and osteoarthritis [258] models. Based on the published results, it can be concluded that CCR3-targeted therapy may be beneficial since its antagonist positively modulates immune cell activation and influx in neuropathic pain. Due to these promising results, we believe that the role of CCR3 in neuropathy should be further explored; moreover, novel pharmacological tools are needed.

#### 2.1.4. CCR4

CCR4 has two selective ligands, CCL17 and CCL22, and one nonselective ligand, CCL2, which all have strong pronociceptive effects. Although the well-known pronociceptive properties of CCL2 are primarily associated with CCR2 [9,52,202], recent research provides evidence that CCL2/CCR4 signaling is also involved in these effects [80]. CCR4 is present on many immune cells, including T lymphocytes (Th2, Th17, and Tregs), platelets, natural killer (NK) cells, macrophages, dendritic cells [127,128,259], neurons [260], microglia [244], and astroglia [128,244]. Importantly, CCR4 is localized at different levels of the PNS (DRGs [103]) and CNS (spinal cord [261], brain [262]), which suggests its crucial role in nociceptive transmission. It was already shown that both of its selective ligands are enhanced in the serum of patients with fibromyalgia [122]. For pharmacological studies, several CCR4 antagonists are available [263,264]; however, the best studied thus far in neuropathic pain models is C021. It has been shown that single and repeated intrathecal and intraperitoneal injections of C021 diminish hypersensitivity in rats and/or mice with CCI- [27,80] and STZ-evoked [79] neuropathic pain. Importantly, C021 in the STZ model also improves locomotor activity [79], which is important from a clinical point of view since patients with diabetes mellitus often experience a decline in locomotor performance and neuropathic pain [265,266]. Surprisingly, in the CNS, the spinal levels of *CCL17* and *CCL22* in rats and mice after nerve injury are unchanged [27,80], similar to diabetic neuropathy [79]. Nevertheless, an increase in *CCL2* in the spinal cord is observed in neuropathic pain models [53,79]. These results highlight the important role of the spinal CCL2/CCR4 axis in nociception. Additionally, it is worth noting that the repeated intrathecal administration of C021 reduces hypersensitivity and, in parallel, diminishes the spinal levels of macrophage/microglial activation, influx, and/or proliferation and, as a consequence, the expression of the pronociceptive IL-1beta and IL-18 [27]. In the PNS, all endogenous CCR4 ligands are elevated in DRGs. Therefore, CCR4 blockade is likely more effective when C021 is administered intraperitoneally, which is not similar to other chemokine antagonists, such as RS5043930 (antagonist of CCR2) [60] or maraviroc (antagonist of CCR5) [206]. Moreover, repeated intraperitoneal treatment with C021 diminished spinal macrophage/microglia levels during neuropathy development [80]. Therefore, targeting CCR4 is a promising strategy to provide a new basis for understanding neuropathic pain pathomechanisms with potentially new therapeutic utility. Importantly, in CCI-exposed rats and mice, the single, intrathecal, and intraperitoneal administration of C021 enhances the analgesic effect of morphine and buprenorphine [27,80] and diminishes the development of morphine tolerance in mice after nerve injury [80]. Overall, published data indicate that targeting CCR4 and its ligands is a promising strategy to provide neuropathic pain relief and enhance the analgesic effects of opioids, which represents a promising basis for the development of more effective combined therapy for pain treatment. Importantly, clinical trials have been studying the use of mogamulizumab, a monoclonal antibody against CCR4, and the results are promising for the treatment of lymphomas and leukemia [267,268] and advanced solid tumors [268], and perhaps one day, it may be explored for neuropathic pain relief.

#### 2.1.5. CCR5

In 2013, Lee et al. [255] showed that CCR5-knockout mice develop hypersensitivity to a lesser extent, which, for the first time, drew attention to the possible involvement of this receptor in nociceptive transmission. It is now known that many endogenous CCR5 ligands exhibit strong pronociceptive effects. CCL3, CCL5, CCL7, CCL8, and CCL11 were described previously. CCR5 is expressed by a variety of immune cells (e.g., granulocytes, macrophages, and lymphocytes) but also in neurons and glia, including astroglia and microglia [269,270]. CCR5 is strongly upregulated in the ipsilateral dorsal spinal cord and DRGs after CCI, and the intrathecal administration of maraviroc (CCR5 antagonist) prevents these changes and, in parallel, the activation of microglia/macrophages and astroglial cells, which are known to be responsible for hypersensitivity development in neuropathy. The observed beneficial effect of maraviroc on CCI-evoked cell activation contributes to the reduction in secreted pronociceptive factors, such as IL-1β, IL-18, IL-6, nitric oxide synthase 2, CCL3, CCL4, and CCL5 [56,206], and in parallel, it is responsible for increasing the antinociceptive factors IL-1 receptor antagonist, IL-18 binding protein, and IL-10 [206]. It was also shown that another CCR5 antagonist, TAK-779, reduces microglial/macrophage activation/migration [271,272]. The obtained published data allow for the hypothesis that maraviroc attenuates neuropathy symptoms by promoting spinal glial “alternative” polarization and restoring the balance between pro- and antinociceptive factors. Despite numerous studies, the detailed mechanism of CCR5 action has not yet been fully ascertained, similar to the reason for the decreased analgesic potency of opioids in neuropathy. Interestingly, opioid receptor agonists, such as morphine, can increase CCR5 expression [273]. Therefore, it was suggested that the heterologous desensitization of opioid and chemokine receptors is possible, since it is already known that CCR5 and opioid receptors (MOR, KOR) are present on spinal glial and neuronal cells [101,273,274,275]. Currently, it is well established that MOR-CCR5 forms heterodimers, which contribute to cross-desensitization [276,277]. Therefore, it is not surprising that the observed maraviroc-evoked downregulation of CCR5, which is upregulated after CCI, beneficially influences opioid agonist effectiveness. Moreover, maraviroc diminishes microglial and astroglial cell activation, and in consequence, these cells secrete fewer factors whose anti-opioid role has already been documented, such as IL-1β [225], IL-18 [224], CCL3 [15,19,22], and CCL5 [25,26,100]. Importantly, data in the literature indicate that other CCR5 antagonists, i.e., AZD5672 and TAK-220, similar to maraviroc, diminish CCI-evoked neuropathic pain symptoms and enhance morphine-evoked analgesia [57]. Numerous studies clearly indicate that CCR5 is a potential target for drug development in the treatment of neuropathic pain, and maraviroc seems to be a substance worth future research in the clinic. Importantly, maraviroc has received accelerated approval from the Food and Drug Administration for clinical use and is currently used as a cure for HIV-infected patients who are infected by the R5-tropic virus [278,279], which indicates that the substance is safe for patients. In our opinion, maraviroc may, in the future, become a drug for the concomitant treatment of patients receiving opioid therapy for neuropathic pain.

#### 2.1.6. CCR8

The role of CCR8 in nociception processes is poorly known thus far, while much is already known about the strong pronociceptive properties of its selective ligand, CCL1 [14]. Moreover, its increase has already been shown at the spinal cord and DRG levels in neuropathy of various etiologies. To date, it has not been demonstrated whether and to what extent the blockade of this receptor affects neuropathic pain. Four new pharmacological tools (AZ084; R243; type 1 and type 2 CCR8 antagonists) have recently been synthesized and are already enabling research. To date, it has been shown that nerve-ligation-induced hypersensitivity is attenuated in CCR8-knockout mice and that CCR8 siRNA blocks CCI-induced hypersensitivity [14]. Moreover, it was demonstrated by immunofluorescence techniques that at the spinal cord level, CCR8 is present on neurons [13,14]. However, after partial sciatic nerve ligation, the upregulation of CCR8 is observed in both neuronal and glial cells [14]. Later, an in vitro study performed in primary cultures of glial cells confirmed the possible localization of CCR8 in microglial and astroglial cells [13]. CCR8 contributes to neuropathic pain through the spinal release of nociceptive cytokines and is involved in the activation of NMDA receptors [14]. In light of recent evidence, it seems that the CCL1/CCR8 axis also plays an important role in opioid effectiveness during neuropathic pain, since neutralizing antibodies against CCL1 enhance the effects of morphine and buprenorphine [13]. Published data indicate that CCL1/CCR8 crosstalk contributes to the development of neuropathic pain of different etiologies [13,14] and, therefore, can be a potential target for drug development in the treatment of neuropathic pain; however, pharmacological studies with newly synthesized antagonists are still needed.

To sum up, clinical data clearly indicate the potential of already-created CC receptor blockers that are being used or currently tested for the treatment of various diseases—e.g., CCR1 antagonists (MLN3897, CP-481,715, AZD-4818, BAY86-5047) [233,234], a CCR2 antagonist (BMS-741672) [239], a monoclonal antibody against CCR4 [267,268], and a CCR5 antagonist (maraviroc) [278,279]. Experimental data suggest that in the future, they may also be used for neuropathic pain relief, but this requires clinical trials. 

### 2.2. Analgesic Potential of Targeting Single CXC Chemokine Receptors

Literature data indicate that the blockade of four and activation of one of the CXC receptors cause analgesia, and two of them additionally improve the effectiveness of opioids in different neuropathic pain models (Table 4).

#### 2.2.1. CXCR2

**CXCR2** has three selective (CXCL1–3) and four unselective (CXCL5–8) endogenous ligands. In neuropathy, CXCR2 is upregulated at the spinal cord level in neuronal [31,32] and nonneuronal [32,287,288] cells and locally at the nerve injury site in macrophages and neutrophils [33]. The spinal neuronal expression of CXCR2 correlates well with the fast and strong pronociceptive effects of intrathecally injected CXCR2 ligands in naive mice [31]. In 2017, it was shown that the administration of the dual CXCR2/CXCR1 antagonist SCH527123 potently reverses central sensitization after brain injury [289]. Recently, in 2019, it was shown for the first time that a potent and selective CXCR2 receptor antagonist, NVP-CXCR2-20, administered intrathecally for seven days reduced the symptoms of neuropathic pain and the CCI-upregulated levels of CXCL3 in the spinal cord and DRGs. Moreover, in naive mice, this antagonist prevents CXCL3-induced hypersensitivity [31]. In addition, the repeated intrathecal administration of NVP-CXCR2-20 reduces the spinal secretion of pronociceptive interleukins (i.e., IL-1beta, IL-6, IL-18) and chemokines CCL2, CCL6, CCL7, and CXCL4 [58]. It was also shown that another CXCR2 antagonist, SB225002, decreases pain symptoms in many animal pain models, such as those induced by paclitaxel [280], SNL [32], L5-SNL [282], PSL [33], CCI [290], and inferior alveolar nerve transection [281]. Moreover, vincristine induces CXCR2 upregulation in spinal cord neurons, which is diminished by levo-corydalmine, consequently leading to a decrease in pain hypersensitivity [291]. However, the repeated intrathecal administration of NVP-CXCR2-20 does not improve the analgesic efficacy of morphine and buprenorphine in a CCI model [31]. According to the authors, this is because the blockade of CXCR2 does not affect microglial or astroglial cell activation [31], as has been observed with antagonists of other chemokine receptors and has already been described in our review. Based on published data, we suggest that the blockade of CXCR2 signaling may have effective analgesic effects in neuropathy [292]. Nevertheless, the lack of effect on opioid drug analgesia indicates that the blockade of other chemokine receptors may be more beneficial in the polytherapy of neuropathy.

#### 2.2.2. CXCR3

Many published studies indicate that CXCR3 is an essential target for the development of neuropathic pain [28,58,143,293]. For the first time, in 2017, hypersensitivity was shown to be markedly reduced in CXCR3-knockout mice, and spinal inhibition of CXCR3 with shRNA attenuated fully developed neuropathic pain after SNI [163]. Moreover, CXCR3 knockout in animals alleviates trigeminal neuropathic pain [165]. To date, five CXCR3 ligands have been identified—CXCL4, CXCL9, CXCL10, CXCL11, and CCL21—and all of them have strong pronociceptive properties [28,294,295]. Recently, it was shown that after CCI [58,295] and/or SNI [143], the spinal level of CXCR3 is upregulated in parallel with its endogenous ligands [28]. Spinal CXCR3 is predominantly expressed on neuronal cells, rarely on microglia, and has not been detected in astrocytes [163]. The mainly neuronal location of CXCR3 confirms and explains why intrathecally administered CXCR3 ligands induce fast and strong pain-like behaviors in naive mice [28]. The binding of ligands to CXCR3 is associated with an increase in the intracellular Ca^2+^ concentration and the activation of MAPKs and NF-κB, which, as is well known, contributes to the development of hypersensitivity [296,297,298,299]. Therefore, the single and repeated intrathecal administration of a potent and selective CXCR3 antagonist ((±)-NBI-74330) attenuates neuropathic pain symptoms. Studies are needed to examine its molecular mechanisms of action. However, it is already known that the repeated administration of (±)-NBI-74330 significantly diminishes microglial cell activation and downregulates the expression of most of its ligands (CXCL4, CXCL9, CXCL10, and CCL21) in the spinal cord of rats with neuropathic pain symptoms. Importantly, behavioral experiments have shown that a CXCR3 antagonist is able to restore the analgesic properties of morphine. The exact role of CXCR3 ligands in opioid effectiveness requires more studies; however, it is already known that the inhibition of microglial activation and the secretion of nociceptive factors by pharmacological tools improve opioid analgesia [222,300]. Additionally, it was recently shown that CXCR3 colocalizes with neurons in the anterior cingulate cortex, and its CCI-evoked upregulation arises in parallel with hypersensitivity development. Importantly, the pharmacological blockade of CXCR3 using an injection of AMG487 directly into the anterior cingulate cortex reduces pain symptoms [283]. In sum, the available literature supports the theory that CXCR3 represents a novel strategy for effective neuropathic pain therapy, including polytherapy with opioids.

#### 2.2.3. CXCR4

AMD3100 is a CXCR4 antagonist that has already been used in the clinic [301]. AMD3100 shows potential therapeutic effects on many inflammatory diseases [302,303,304] and is approved by the FDA for autologous stem cell transplantation in patients with non-Hodgkin’s lymphoma and multiple myeloma [301]. Moreover, AMD3100 causes pain relief in AIDS-induced neuropathy [305]. Animal studies have shown that AMD3100 has analgesic effects on different pathological pain states in mice and rats, including bone cancer pain [172,306], diabetic neuropathy [307,308], peripheral neuropathic pain [37,284,309], and opioid-induced hyperalgesia [310]. Another CXCR4 antagonist, AMD3465, exhibits analgesic properties in animal models, such as pSNL and CPIP models [37]. In the SNL-induced rat neuropathic pain model, CXCR4 and its endogenous ligand CXCL12 are highly upregulated, mainly in astroglia and neuronal cells. Studies have also shown that CXCR4 colocalizes with microglia from the spinal dorsal horn. Moreover, the inhibition of CXCL12 and the blockade of CXCR4 relieve neuropathic pain symptoms [39]. AMD3100 alleviates hypersensitivity partially by augmenting leukocyte-derived endogenous opioid secretion in the spinal cord and downregulating pronociceptive factors (TNFα, IL-1β) [284]. Importantly, local naloxone methiodide injection inhibits AMD3100-related analgesia [284]. Moreover, the intraperitoneal administration of AMD3100 dose-dependently reverses opioid-induced hyperalgesia in rats [310]. Published papers provide evidence that analgesia via CXCR4 is mediated by the endogenous opioid system [284]. In summary, data from the literature suggest that the blockade of CXCR4 signaling represents a promising novel approach to the management of side effects associated with the use of opioids for chronic pain management [310]; however, studies in neuropathic pain models are needed.

#### 2.2.4. CXCR8

In 2015, it was shown that CXCR8, also known as GPR35, is a receptor for CXCL17 [184]; however, the role of the CXCL17/CXCR8 axis in nociceptive transmission still needs to be studied. CXCR8 expression has been identified within the nervous system in both neuronal and nonneuronal cells, including glia, and is therefore suggested to be important for neuropathic pain development [311,312,313,314,315,316]. However, kynurenic acid is also an important agonist of CXCR8 [317,318,319]. It has already been shown that the intrathecal administration of CXCL17 in naive mice induces pain-related behaviors, which, however, are partially abolished by the administration of kynurenic acid. Moreover, in contrast to CXCL17, kynurenic acid diminishes pain-related behavior evoked by nerve injury and enhances the effectiveness of morphine [42,286]. However, kynurenic acid is not only an agonist of CXCR8 but also a noncompetitive antagonist at the glycine site of the NMDA receptor and an antagonist of ionotropic NMDA, AMPA, and kainate receptors [314,320,321,322,323]. Considering the abovementioned multitarget effects of kynurenic acid, it is not surprising that it has analgesic properties in a mouse/rat pain model [313,315,319,324,325]. However, similar effects were observed with zaprinast, an exogenous agonist of CXCR8 [316], which also partly diminished CXCL17-evoked hypersensitivity in naive mice [42]. Moreover, its analgesic properties have been proven in rat and mouse inflammatory [313,326] and neuropathic [42,286] pain models. To date, it has been shown that the activation of CXCR8 by zaprinast, kynurenic acid, or CXCL17 leads to the distinct modulation of Ca^2+^ levels: kynurenic acid and zaprinast reduce Ca^2+^ mobilization [327], while in contrast, CXCL17 increases Ca^2+^ mobilization [184]. Recently, two in vitro studies published by Binti et al. [185] and Park et al. [328] proposed that CXCL17 also acts through an unknown receptor. This is in line with published behavioral studies showing that the pronociceptive properties of CXCL17 are not completely blocked by zaprinast and kynurenic acid. Published data suggest that CXCR8 is a promising target for reducing neuropathic pain and enhancing opioid analgesia, but this hypothesis requires further in-depth research.

In sum, although pharmaceutical companies put a lot of effort into the production of CXC receptors antagonists, despite good results obtained in experimental studies in general only substances targeting CXCR4 are commonly used in clinical trials [329]. 

### 2.3. Analgesic Potential of Targeting the Single XCR Chemokine Receptor

Data in the literature indicate that the blockade of **XCR1** causes analgesia and improves the effectiveness of opioids in a neuropathic pain model (Table 4). In 2016, it was shown for the first time that the XCL1/XCR1 axis plays an important role in hypersensitivity development in STZ-induced diabetes [44], and in 2022, it was also shown to play an important role in CCI-evoked neuropathy [43]. For a long time, XCR1 was suggested to be the only receptor for XCL1. Nevertheless, since 2017, it has been known that XCL1 also acts via fibroblast migration through the heterodimeric (αβ) transmembrane receptor ITGA9 [193]; thus, it was hypothesized that both of these receptors are involved in the development of neuropathic pain. XCR1 is expressed in neurons [44,330], oligodendrocytes [330], and many immune cells (e.g., T and B cells and neutrophils) [331]. Moreover, immunohistochemical studies have shown that XCR1 is expressed in nonpeptidergic and non-IB4 binding terminals of A-delta and C-fiber afferents and within excitatory interneurons [330]. However, in the spinal cord, the expression of XCR1 was not detected in microglia or astroglia [43]. Behavioral studies have shown that the blockade of XCR1 by vMIP-II may cause neuropathic pain relief [43], which may be due to the ability to reduce the p38MAPK and pERK activation observed after stimulation by XCL1 [330]. Published data indicate that the XCL1/XCR1 axis plays a key role in nociceptive transmission since it is able to participate in many aspects of neuronal–glial interactions.

### 2.4. Analgesic Potential of Targeting a Single CX3C Chemokine Receptor

JMS-17-2 is a new, potent, and selective small-molecule antagonist of **CX3CR1** that is able to inhibit glial cell activation [332]; however, to our knowledge, it has not been studied in neuropathic pain models. Following nerve injury, the spinal enhancement of CX3CR1 levels in microglial cells is observed, together with hypersensitivity development [200,333,334]. Moreover, the intrathecal administration of CX3CL1 evokes both thermal and mechanical hypersensitivity [335]; however, both effects are abrogated in CX3CR1-knockout mice [196,336]. Importantly, it was already shown that a neutralizing antibody against CX3CR1 [337,338] diminishes neuropathic pain symptoms. Additionally, the intrathecal injection of PLGA-encapsulated CX3CR1 siRNA nanoparticles reduces mechanical hypersensitivity in SNL-induced animals [339]. Data in the literature strongly suggest that the CX3CL1/CX3CR1 axis is important for nociceptive transmission [200]; therefore, further studies with the use of novel selective CX3CR1 antagonists are necessary.

### 2.5. Analgesic Potential of Targeting Multiple Chemokine Receptors

Research in recent years has provided an increasing amount of data showing that multifunctional compounds also have good antinociceptive activity. Currently, studies are showing the significant advantages of compounds targeting more than one molecular target over a physical combination of individual substances in terms of their antinociceptive properties, which is the case for multitarget antagonists of chemokine receptors (Table 5), which we will review in the following sections.

UCB35625 is a recently synthesized dual antagonist of **CCR1** and **CCR3 [344]**. The importance of these receptors in nociceptive transmission has already been proven [16,23,53]. It was shown that the blockade of CCR1 by J113863 and CCR3 by SB328437 diminishes pain-like behavior in a CCI model [23,53]. Moreover, it was proven that several pronociceptive chemokines can act through CCR1 and CCR3 [16]. Among them, CCL7 and CCL8 are the common ligands of both receptors with well-known long-lasting increases and strong pronociceptive properties in neuropathy [16]. In 2022, it was shown that UCB35625 diminishes pain-like behavior [16]. However, surprisingly, the reduction in hypersensitivity by this dual agonist of CCR1/3 is similar to or even less than that produced by the single antagonists J113863 and SB328437 administered alone [16]. Nevertheless, we still believe that CCR1 and CCR3 may serve as potential therapeutic targets for the treatment of neuropathic pain, but new and better pharmacological tools that can target several chemokine receptors are needed.

Three dual antagonists of **CCR2** and **CCR5** are currently available: cenicriviroc [55,60], BMS-813160 [345], and, recently, PF-04634817 [346]. To date, only the effectiveness of cenicriviroc has been tested in neuropathic pain models. Many studies highlight the importance of CCR2 and CCR5 in nociceptive transmission [52,56,202,206]. Interestingly, these two receptors are structurally similar: they share 70% sequence homology [8]. Importantly, it has been proven that CCR2 and CCR5 undergo heterodimerization, specifically after costimulation with their ligands [347]. In the CNS, the receptors are coexpressed on infiltrating and/or resident immune (monocytes, lymphocytes) and glial (microglia, astrocytes) cells [8,201,348] and are present on neurons [201]. The common feature of CCR2 and CCR5 ligands is pleiotropy; therefore, some of them have the ability to act through both receptors, and moreover, importantly, most of them evoke pain-related behavior [8,15,19,349]. Recently, it was shown that the repeated intrathecal administration of selective CCR2 (RS504393) [52] and CCR5 (maraviroc) [56] antagonists and a dual CCR2/CCR5 antagonist (cenicriviroc) caused analgesia in a rat neuropathic pain model [55,60]. The authors have shown that both single and dual antagonists similarly diminish the activation and infiltration of microglia and macrophages in the spinal cord and/or DRGs and lower the levels of pronociceptive interleukins, such as IL-1beta, IL-6, and IL-18 [52,55,56]. However, the dual antagonist is a more effective agent in silencing a broad spectrum of chemokines. It was shown that the administration of cenicriviroc results in the downregulation of many pronociceptive chemokines, including *CCL2*, *CCL3*, *CCL4*, *CCL5*, *CCL7*, and *CCL12*, in DRGs and/or the spinal cord. In contrast, single antagonists affect the biosynthesis of pronociceptive chemokines to a lesser extent; RS504393 reduces the expression of *CCL2*, *CCL3*, *CCL4*, *CCL5*, *CCL7*, and *CCL11* only in DRGs, whereas maraviroc diminishes only the level of *CCL5* in DRGs and *CCL4* in the spinal cord. Undoubtedly, the dual antagonist cenicriviroc is the most effective drug for silencing pronociceptive chemokine signaling pathways. Moreover, importantly, from a clinical point of view, the intraperitoneal injection of cenicriviroc has greater analgesic properties than RS504393 or maraviroc against neuropathic pain [60]. In addition, it is also extremely significant that repeated intrathecal and intraperitoneal injections of cenicriviroc strongly enhance the analgesic effects of morphine and buprenorphine [55,60]. The mechanism underlying this phenomenon is not clear; however, it may be related to heterologous desensitization between opioid/chemokine receptors or a beneficial influence on neuroimmune interactions [60], or probably both processes. This is clinically relevant, particularly since cenicriviroc is a drug candidate for the treatment of HIV infection, nonalcoholic steatohepatitis, and liver fibrosis (CENTAUR, phase 2b, and AURORA, phase 3) [350]. Based on data in the literature, it seems that targeting CCR2 and CCR5 simultaneously is an interesting alternative for neuropathic pain pharmacotherapy, especially in combination with opioids. However, further behavioral and clinical research is needed.

In 2004, reparixin (also called repertaxin), a potent **CXCR1** and **CXCR2** allosteric inhibitor, was presented for the first time [351]. Later, it was shown that this substance diminishes the development of paclitaxel-induced nociception in rats [342]. In 2023, it was shown that reparixin blocks oxaliplatin-induced hypersensitivity but not vincristine-induced hypersensitivity [343]. Considering that CXCR1 and CXCR2 are upregulated in several animal models of neuropathic pain [282,352], it is undoubtedly important to conduct further research with reparixin.

Another interesting pharmacological tool is RAP-103, which blocks four chemokine receptors, **CCR2**, **CCR5**, **CCR8** [353], and **CXCR4**, as the latest research shows [354]. It was already shown that RAP-103 prevents and/or diminishes established neuropathic pain hypersensitivity in rats after partial sciatic nerve injury [341] and in a diabetic peripheral neuropathy model [340]. Moreover, RAP-103 evokes analgesia by reducing spinal cord microglial/macrophage activation and monocyte infiltration and by reducing the mRNA expression of IL-1β and IL-6 in the sciatic nerve and spinal cord [341]. Moreover, RAP-103 administered together with morphine enhances its analgesic effect in the DPN model, which is related to the fact that it increases the sensitivity of MOR and reduces the levels of the pronociceptive cytokines TNFα and CCL3, and it also slightly reduced CCL2 [340]. Importantly, in 2022, Bongiovanni et al. reported that RAP-103 diminishes opioid-derived respiratory depression and physical dependence in rats [354]. The authors proposed that this multitarget chemokine receptor antagonist may lessen the risk of opioid abuse [354], which indicates that polytherapy may be useful for the management of both pain and opioid addiction. Therefore, in light of the current literature, it can be said with certainty that by blocking several chemokine receptors that are significantly involved in nociceptive transmission, pain can be relieved; such therapeutic approaches may be extremely helpful in wide range of clinical applications, including opioid pharmacotherapy.

## 3. Conclusions

A deeper understanding of the role of chemokines and their temporal fluctuations is crucial for the development of novel therapeutic strategies for neuropathy. Data in the literature clearly indicate that numerous chemokines possess pronociceptive effects; therefore, blocking their action by using neutralizing antibodies or blocking their biosynthesis relieves pain. For example, from the CC family, CCL1/2/3/5/7/8/9/11 are significant in the development (CCL2/3/5/7/8/9) and maintenance (CCL2/7/8) of neuropathic pain.

**Scheme 1:**
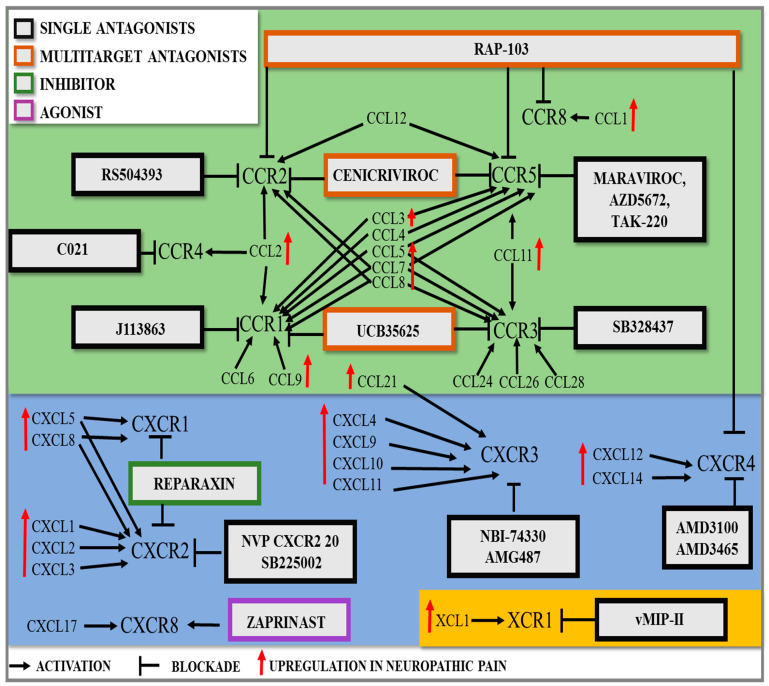
Changes and targets in chemokine systems in animal models of neuropathic pain, prepared based on the following references: [16,19,23,27,28,31,32,33,37,43,53,55,56,57,58,60,79,80,206,280,281,282,283,284,285,330,340].

Among them, CCL2/7/8 seem to be the most important because of the quick and long-lasting increase in their protein levels and their strong pronociceptive properties. Additionally, chemokines from the CXC family, such as CXCL3/9/10/12/13/14/17, from the XC family, such as XCL1, and from the CX3C family, such as CX3CL1, are already known to be crucial during neuropathic pain. The literature data indicate that these chemokines and their receptors are promising targets in the search for novel therapies for neuropathic pain; however, more studies are needed. Many studies, including ours, have shown that the use of single chemokine receptor antagonists (Scheme 1) can relieve pain of different etiologies, for example, the blockade of CCR1 (J113863) [7,8,17], CCR2 (RS504393) [6,187,188,220], CCR3 (SB328437) [7,21], CCR4 (C021) [28,64,65], CCR5 (maraviroc, AZD5672, TAK-220) [15,16,27,190], CXCR2 (NVP CXCR2 20, SB225002) [25,32,34,35,267,268,270], CXCR3 (NBI-74330, AMG487) [25,31,272], CXCR4 (AMD3100, AMD 3465) [43,273,274], and XCR1 (vMIP-II) [52,276]. It has been shown that some chemokines, e.g., CCL1 [5], CCL2 [12], CCL3 [17], CCL7 [12], CCL9 [17], and XCL1 [52], reduce the analgesic effect of opioids, and the use of neutralizing antibodies restores morphine and/or buprenorphine analgesia.

**Scheme 2:**
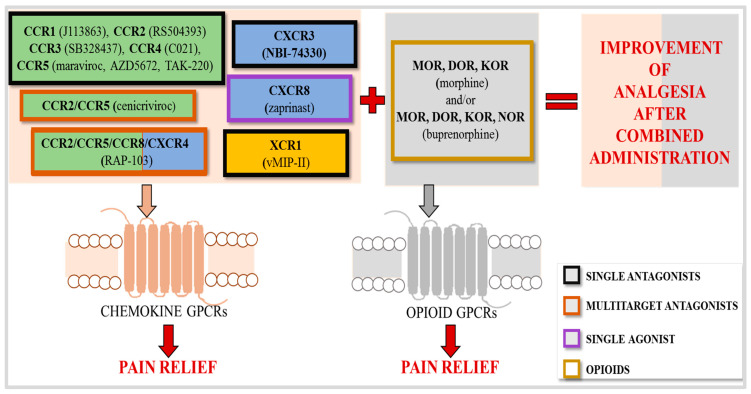
Targeting chemokine systems enhances opioid effectiveness, prepared based on the following references: [19,23,27,28,31,43,52,53,55,56,58,60,79,80,330,340,342].

Similarly, single antagonists of chemokine receptors, e.g., J113863 [19,53], RS504393 [52], C021 [27,79,80], maraviroc [56,60], vMIP-II [43,330], and NBI-74330 [28,31,58], enhance opioid analgesia due to their ability to inhibit the activation of microglia/macrophages (Scheme 2). The exception is SB328437 [23], which reduces hypersensitivity and enhances opioid analgesia; however, SB328437 does not affect the level of spinal microglial/macrophage activation or infiltration but strongly inhibits the activation/influx of neutrophils in neuropathy [23].

Taking into account the pleiotropy of many chemokines and the different mechanisms of action of single antagonists of chemokine receptors, it seems that pharmacological modulation based on simultaneously blocking several chemokine receptors can be used as an innovative method of treating neuropathic pain, with great effectiveness, and can also be used in combination with opioid drugs. Multitarget chemokine receptor antagonists/inhibitors, such as cenicriviroc (CCR2/5) [55,60], reparixin (CXCR1/2) [342], and RAP-103 [340] (CCR2/5/8/CXCR4) (Scheme 1), seem to be interesting painkillers. Such a multidirectional treatment strategy based on the modulation of neuronal–glial–immune interactions by changing the activity of the chemokine family can significantly improve the quality of life of patients suffering from neuropathic pain.

## Figures and Tables

**Table 1:** Chemokine family—ligands and their receptors, their pronociceptive properties and chemokine-neutralizing antibody effectiveness: evidence from mouse and/or rat studies.

Chemokine	Chemokine Receptors	Chemokine-Evoked Hypersensitivity	nAb against Chemokine-Evoked Analgesia
**CCL1**, I-309	**CCR8**	**YES** [13,14]	**PSNL** [14], **STZ** [13]
**CCL2**, MCP-1	**CCR1, CCR2, CCR4**	**YES** [15,16,17]	**CCI** [15], **OX** [18]
**CCL3**, MIP-1α	**CCR1, CCR5**	**YES** [16,19,20,21]	**STZ** [19], **PTX** [22], **CCI** [15,23], **PSNL** [21]
**CCL4**, MIP-1β	**CCR1, CCR5**	**YES** [19]	**PSNL** [24]
**CCL5**, RA—NTES	**CCR1, CCR3, CCR5**	**YES** [16]	**L5Tx** [25], **CCI** [26]
**CCL6, -**	**CCR1**	**ND**	**ND**
**CCL7,** MCP3	**CCR1, CCR2, CCR3, CCR5**	**YES** [15,16]	**CCI** [15]
**CCL8,** MCP-2	**CCR1, CCR2, CCR3, CCR5**	**YES** [16]	**ND**
**CCL9/,** MIP-1γ, **CCL10**	**CCR1**	**YES** [16,19]	**STZ** [19]
**CCL11**, Eotaxin	**CXCR3, CCR3, CCR5**	**ND**	**ND**
**CCL12**, MCP-5	**CCR2**	**NO** [15]	**ND**
**CCL13**, MCP-4	**CCR1, CCR2, CCR3, CCR5**	**NOT PRESENT IN MICE/RATS**	
**CCL14**, HCC-1	**CCR1**	**NOT PRESENT IN MICE/RATS**	
**CCL15**, HCC-2	**CCR1, CCR3**	**NOT PRESENT IN MICE/RATS**	
**CCL16**, HCC-4	**CCR1**	**NOT PRESENT IN MICE/RATS**	
**CCL17**, TARC	**CCR4**	**YES** [27]	**ND**
**CCL18**, DC-CK1	**CCR8**	**NOT PRESENT IN MICE/RATS**	
**CCL19**, MIP-3β	**CCR7**	**ND**	**ND**
**CCL20**, MIP-3α	**CCR6**	**ND**	**ND**
**CCL21**, 6Ckine	**CXCR3, CCR7**	**YES** [28,29]	**CCI** [28] SNI [30] BTZ [29]
**CCL22**, MDC	**CCR4**	**YES** [27]	**ND**
**CCL23**, MPIF-1	**CCR1**	**NOT PRESENT IN MICE/RATS**	
**CCL24**, Eotaxin-2	**CCR3**	**ND**	**ND**
**CCL25**, TECK	**CCR9, CCR10**	**ND**	**ND**
**CCL26**, Eotaxin-3	**CCR3, CCR10**	**ND**	**ND**
**CCL27**, CTACK	**CCR10**	**ND**	**ND**
**CCL28,** MEC	**CCR3, CCR10**	**ND**	**ND**
**CXCL1**, GROα, CINC-1	**CXCR2**	**YES** [31]	**SNL** [32]
**CXCL2**, GROβ, CINC-3	**CXCR2**	**YES** [31]	**PSNL** [33]
**CXCL3**, GROγ, CINC-2	**CXCR2**	**YES** [31]	**CCI** [31]
**CXCL4**, PF4	**CXCR3**	**YES** [28]	**ND**
**CXCL5**, ENA-78	**CXCR1, CXCR2**	**YES** [34]	**CCI** [34]
**CXCL6**, GCP-2	**CXCR1, CXCR2**	**ND**	**ND**
**CXCL7**, NAP-2	**CXCR1, CXCR2**	**ND**	**ND**
**CXCL8**, IL-8	**CXCR1, CXCR2**	**ND**	**ND**
**CXCL9**, Mig	**CXCR3**	**YES** [28]	**CCI** [28]
**CXCL10**, IP-10	**CXCR3**	**YES** [28,35]	**CCI** [28], **CIBP** [36]
**CXCL11**, I-TAC	**CXCR3**	**YES** [28]	**ND**
**CXCL12**, SDF-1α/β	**CXCR4**	**YES** [37]	**SpNI** [38], **SNL** [39]
**CXCL13**, BCA-1	**CXCR5**	**YES** [40]	**CPIP** [41]
**CXCL14**, BRAK	**CXCR4**	**ND**	**ND**
**CXCL15**, Lungkine	**Unknown**	**ND**	**ND**
**CXCL16**, SCYB16	**CXCR6**	**ND**	**ND**
**CXCL17**, VCC-1	**CXCR8 (GPR35)**	**YES** [42]	**ND**
**XCL1**, SCM-1α	**XCR1**	**YES** [43,44]	**CCI** [43] **STZ** [44]
**CX3CL1**, Fractalkine	**CX3CR1**	**YES** [45]	**OX** [46]

Abbreviations: PSNL—partial sciatic nerve ligation; STZ—streptozotocin-evoked neuropathic pain; CCI—chronic constriction injury; OX—oxaliplatin-induced neuropathic pain; PTX, paclitaxel-induced neuropathic pain; L5Tx—spinal nerve L5 transection; SNI, spinal nerve injury; BTZ—bortezomib-evoked neuropathic pain; CIBP—cancer-induced bone pain; SCI—spinal cord injury; SpNI—spared nerve injury; SNL—spinal nerve ligation; CPIP—chronic postischemic pain; ND—no data available.

**Table 2:** The central and peripheral nervous system upregulation of chemokines at the mRNA and protein levels in neuropathic pain models: evidence from mouse and/or rat studies.

Chemokine	Upregulation in Neuropathic Pain Models			
	mRNA	Structures/Models	Protein	Structures/Models
**CCL1**	**YES**	**SC, DRG/PSNL** [14], **DRG/CCI** [50]	**YES**	**SC/PSNL** [14], **SC/STZ** [13]
**CCL2**	**YES**	**DRG/CCI** [50,51], **SC, DRG/CCI** [52,53], **SC/CCI** [16]	**YES**	**SC, DRG/CCI** [52], **SC/CCI** [16], **DRG/CCI** [51], **DRG/OX** [18], **SC/SCI** [54]
**CCL3**	**YES**	**SC, DRG/CCI** [55,56], **SC/CCI** [53,57]	**YES**	**SC/CCI** [16,57], **SC/STZ** [19]
**CCL4**	**YES**	**SC/CCI** [16,53,57]	**NO**	**SC/CCI** [16]
**CCL5**	**YES**	**SC/CCI** [16]	**YES**	**SC/CCI** [16,26]
**CCL6**	**YES**	**SC, DRG/CCI** [53,58], **SC/CCI** [16]	**NO**	**SC/CCI** [16]
**CCL7**	**YES**	**SC, DRG/CCI** [23,53,58], **SC/CCI** [16], SC/PSNL [59]	**YES**	**SC, DRG/CCI** [23], **SC/CCI** [16]
**CCL8**	**YES**	**SC/CCI** [16]	**YES**	**SC/CCI** [16]
**CCL9**	**YES**	**SC, DRG/CCI** [53], **SC/CCI** [16,57]	**YES**	**SC/CCI** [16,57], **SC/STZ** [19]
**CCL11**	**YES**	**SC, DRG/CCI** [23]	**YES**	**DRG/CCI** [23]
**CCL12**	**YES**	**SC, DRG/CCI** [60] SC [15]	**ND**	**ND**
**CCL13**	**NOT PRESENT IN MICE/RATS**			
**CCL14**	**NOT PRESENT IN MICE/RATS**			
**CCL15**	**NOT PRESENT IN MICE/RATS**			
**CCL16**	**NOT PRESENT IN MICE/RATS**			
**CCL17**	**NO**	**SC/CCI** [27]	**ND**	**ND**
**CCL18**	**NOT PRESENT IN MICE/RATS**			
**CCL19**	**ND**	**ND**	**ND**	**ND**
**CCL20**	**ND**	**ND**	**ND**	**ND**
**CCL21**	**YES**	**SC/BTZ** [29]	**YES**	**SC/SNI** [61], **DRG/SNI** [30], **SC/BTZ** [29]
**CCL22**	**NO**	**SC/CCI** [27]	**ND**	**ND**
**CCL23**	**NOT PRESENT IN MICE/RATS**			
**CCL24**	**NO**	**SC, DRG/CCI** [23], **SC/CCI** [16]	**ND**	**ND**
**CCL25**	**ND**	**ND**	**ND**	**ND**
**CCL26**	**YES**	**SC/CCI** [23]	**ND**	**ND**
**CCL27**	**ND**	**ND**	**ND**	**ND**
**CCL28**	**NO**	**SC/CCI** [16]	**ND**	**ND**
**CXCL1**	**YES**	**SC/SNL** [32], **SC/STZ** [62], **SC/CCI** [31]	**YES**	**SC/SNL** [32], **SC/STZ** [62]
**CXCL2**	**YES**	**SC/CCI** [31], **SCN/PSNL** [33], **SN/STZ** [63]	**YES**	**SN/STZ** [63]
**CXCL3**	**YES**	**SC/CCI** [31]	**YES**	**SC, DRG/CCI** [31]
**CXCL4**	**YES**	**SC, DRG/CCI** [28]	**YES**	**SC, DRG/CCI** [28]
**CXCL5**	**YES**	**SC/CCI** [34]	**YES**	**SC/STZ** [62]
**CXCL6**	**ND**	**ND**	**NO**	**CSF/CCI** [64]
**CXCL7**	**ND**	**ND**	**ND**	**ND**
**CXCL8**	**YES**	**DRG/LDH** [65]	**YES**	**SN/CCI** [66]
**CXCL9**	**YES**	**DRG/CCI** [28] **SC/STZ** [62], **DRG/SNL** [67]	**YES**	**SC, DRG/CCI** [28], **SC/STZ** [62]
**CXCL10**	**YES**	**SC/CCI** [28], **DRG/SNL** [67]	**YES**	**SC/CCI** [28,35]
**CXCL11**	**YES**	**SC, DRG/CCI** [28], **DRG/SNL** [67,68], **SC/STZ** [62]	**YES**	**SC/CCI** [28]
**CXCL12**	**YES**	**DRG/CCD** [69]	**YES**	**SC/STZ** [62], **SC/SNL** [39], **SC/PSNL/CPIP** [37], **SC/SpNI** [38], **ACC/STZ** [70]
**CXCL13**	**YES**	**SC/SNL** [40], **SC/DB** [71], **DRG/SNL** [68,72], **DRG/SpNI** [73], **SC/TNFα** [74]	**YES**	**SC/SNL** [40], **SC/DB** [71], **SC**, **DRG,PS/CCI** [75], **SC/TNFα** [74]
**CXCL14**	**YES**	**SC/PTX** [76], **DRG/SNL** [68]	**YES**	**SC/PTX** [76]
**CXCL15**	**ND**	**ND**	**ND**	**ND**
**CXCL16**	**ND**	**ND**	**ND**	**ND**
**CXCL17**	**ND**	**ND**	**ND**	**ND**
**XCL1**	**YES**	**SC/CCI** [43]	**YES**	**SC/CCI** [43], **SC/STZ** [44]
**CX3CL1**	**YES**	**DRG/OX** [46], **SC/TNFα** [74]	**YES**	**DRG/OX** [46,77], **DRG/CCI** [77], **SC/TNFα** [74]

Abbreviations: PSNL—partial sciatic nerve ligation; CCI—chronic constriction injury; STZ—streptozotocin-evoked neuropathic pain; OX—oxaliplatin-induced neuropathic pain; SCI—spinal cord injury; BTZ—bortezomib-evoked neuropathic pain; SNI—spinal nerve injury; SNL—spinal nerve ligation; LDH—lumbar disc herniation; CCD—chronic compression of the dorsal root ganglion; CPIP—chronic postischemic pain; SpNI—spared nerve injury; DB—diabetes model; TNFα—TNFα-induced; PTX—paclitaxel-induced neuropathic pain; SC—spinal cord; DRG—dorsal root ganglion; SCN—common sciatic nerve; SN—sciatic nerve; CSF—cerebrospinal fluid; ACC—anterior cingulate cortex; ND—no data available.

**Table 3:** Analgesic potential of targeting single CC chemokine receptors in animal models of neuropathic pain.

Receptor	Antagonist	Neuropathic Pain Relief in Mouse/Rat Models/Route of Drug Administration	Enhancement of Opioid Effectiveness in Neuropathy
**The CC Receptors**			
**CCR1**	**J113863**	**CCI/i.t.** [16,53] **STZ/i.t** [19]	**YES** [19,53] **ND**
	**BX513**	**SSNL/i.t.** [20]	**ND**
	**BX471**	**ND**	**ND**
	**BAY86-5047**	**ND**	**ND**
**CCR2**	**RS504393**	**CCI/i.t.** [52,202] **PTX/s.c.** [203] **IAMNT/ic**. [204]	**YES** [52] **ND** **ND**
	**AZ889**	**CCI/ p.o.** [205]	**ND**
	**INCB 3284**	**ND**	**ND**
	**RS 102895**	**ND**	**ND**
	**MK-0812**	**ND**	**ND**
	**PF-4136309**	**ND**	**ND**
**CCR3**	**SB328437**	**CCI/i.t.** [16,23]	**YES** [23]
	**SB 297006**	**ND**	**ND**
	**GW 766994**	**ND**	**ND**
	**INCB 3284**	**ND**	**ND**
**CCR4**	**C021**	**CCI/it./i.p.** [27,80] **STZ/i.t/i.p.** [79]	**YES** [27,80] **YES** [79]
	**AZD 2098**	**ND**	**ND**
	**GSK 2239633A**	**ND**	**ND**
**CCR5**	**Maraviroc**	**CCI/i.t.**/i.p. [56,60,206]	**YES** [56,60]
	**AZD5672**	**CCI/i.t.** [57]	**YES** [57]
	**TAK-220**	**CCI/i.t.** [57]	**YES** [57]
	**Vicriviroc**	**ND**	**ND**
**CCR6**	**PF-07054894**	**ND**	**ND**
**CCR7**	**-**	**ND**	**ND**
**CCR8**	**AZ084**	**ND**	**ND**
	**R243**	**ND**	**ND**
**CCR9**	**Vercirnon**	**ND**	**ND**
	**MLN3126**	**ND**	**ND**
**CCR10**	**BI-6901**	**ND**	**ND**

Abbreviations: CCI—chronic constriction injury of the sciatic nerve model; STZ—streptozotocin-induced diabetic model; SSNL—segmental spinal nerve ligation model; IAMNT—inferior alveolar nerve and mental nerve transection model; PTX—paclitaxel-induced peripheral neuropathic pain model; ND—no data available; i.t.—intrathecal; s.c.—subcutaneous; ic.—intracisternal, p.o.—per os; i.p.—intraperitoneal.

**Table 4:** Analgesic potential of targeting the single CXC, XC, and CX3 receptors in animal models of neuropathic pain.

Receptor	Antagonist/ Agonist	Neuropathic Pain Relief in Mouse/Rat Models	Enhancement of Opioid Effectiveness in Neuropathic Pain
**The CXC Receptors**			
**CXCR1**	-	**ND**	**ND**
**CXCR2**	**NVP CXCR2 20**	**CCI/i.t.** [31,58]	**NO** [31,58]
	**SB225002**	**PTX/i.t/i.p.** [280] **SNL/i.t** [32] **OFP/tg** [281] **PSL/pn** [33] **L5-SNL/ns** [282]	**ND** **ND** **ND** **ND** **ND**
	**SB 332235**	**ND**	**ND**
	**SB 265610**	**ND**	**ND**
	**AZ10397767**	**ND**	**ND**
	**AZD 5069**	**ND**	**ND**
	**Danirixin**	**ND**	**ND**
**CXCR3**	**NBI-74330**	**CCI/i.t.** [28,58]	**YES** [28]
	**AMG487**	**CCI/acc** [283]	**ND**
**CXCR4**	**AMD 3100** **(Plerixafor)**	**L5-SNL/i.p.** [284] **CCD/ive** [285] **CPIP/i.t.** [37]	**ND** **ND** **ND**
	**AMD 3465**	**SNL/i.t.** [37] **CPIP/i.t.** [37]	**ND** **ND**
	**CTCE 9908**	**ND**	**ND**
	**KRH 3955**	**ND**	**ND**
	**IT1t**	**ND**	**ND**
	**WZ811**	**ND**	**ND**
	**Mavorixafor**	**ND**	**ND**
	**BKT 140**	**ND**	**ND**
	**MSX-122**	**ND**	**ND**
**CXCR5**	**-**	**ND**	**ND**
**CXCR6**	**ML-339**	**ND**	**ND**
**CXCR7**	**-**	**ND**	**ND**
**CXCR8**	**Zaprinast**	**CCI/i.t.** [42,286]	**YES** [42,286]
**THE XC RECEPTOR**			
**XCR1**	**vMIP-II**	**CCI/i.t.** [43]	**YES** [43]
**THE CX3C RECEPTOR**			
**CX3CR1**	**JMS 17-2**	**ND**	**ND**

Abbreviations: CCI—chronic constriction injury of the sciatic nerve model; STZ—streptozotocin-induced diabetic model; IAMNT—inferior alveolar nerve and mental nerve transection model; PTX—paclitaxel-induced peripheral neuropathic pain model; SNL—spinal nerve ligation model; PSL—partial sciatic nerve ligation model; L5-SNL—L5 spinal nerve ligation model; OFP model—orofacial pain model; CPIP—chronic postischemia pain; CCD—chronic compression of the DRG model; i.t.—intrathecal; i.p.—intraperitoneal; tg—directly into trigeminal ganglion; pn—perineural; ns—not shown; acc—into anterior cingulate cortex; ive—intervertebral.

**Table 5:** Analgesic potential of targeting multiple multitarget chemokine receptors in animal models of neuropathic pain.

Receptor	Multitarget Antagonist/ Inhibitor	Neuropathic Pain Relief in Mouse/Rat Models	Enhancement of Opioid Effectiveness in Neuropathic Pain
**CCR1/CCR3**	**UCB 35625**	**CCI/i.t.** [16]	**ND**
**CCR2/CCR5**	**Cenicriviroc**	**CCI/i.t./i.p.** [55,60]	**YES** [55]
	**BMS-813160**	**ND**	**ND**
	**PF-04634817**	**ND**	**ND**
**CCR2/CCR5/CXCR3**	**TAK 779**	**ND**	**ND**
**CCR2/CCR5/CCR8/ CXCR4**	**RAP-103**	**STZ/i.p.** [340] **PSNI/p.o.** [341]	**YES** [340]
**CXCR1/CXCR2**	**Reparixin**	**PTX/m.o.p.** [342] **OX/i.t./i.p.** [343]	**ND**
	**Navarixin**	**ND**	**ND**
	**SCH 563705**	**ND**	**ND**
	**Ladarixin**	**ND**	**ND**

Abbreviations: CCI—chronic constriction injury of the sciatic nerve model; STZ—streptozotocin-induced diabetic model; PSNI—partial sciatic nerve injury model; PTX—paclitaxel-induced peripheral neuropathic pain model; OX—oxaliplatin-induced neuropathic pain model; i.t.—intrathecal; i.p.—intraperitoneal; p.o—per os; m.o.p.—micro-osmotic pumps.

## Data Availability

Not applicable.
